# Genetic elements promote retention of extrachromosomal DNA in cancer cells

**DOI:** 10.1038/s41586-025-09764-8

**Published:** 2025-11-19

**Authors:** Venkat Sankar, King L. Hung, Aditi Gnanasekar, Ivy Tsz-Lo Wong, Quanming Shi, Katerina Kraft, Matthew G. Jones, Britney Jiayu He, Xiaowei Yan, Julia A. Belk, Kevin J. Liu, Sangya Agarwal, Sean K. Wang, Anton G. Henssen, Paul S. Mischel, Howard Y. Chang

**Affiliations:** 1 Departments of Dermatology and Genetics, Stanford University, Stanford, CA USA; 2 Sarafan ChEM-H, Stanford University, Stanford, CA USA; 3 Department of Pathology, Stanford University, Stanford, CA USA; 4 Program in Cancer Biology, Stanford University, Stanford, CA USA; 5 Department of Pediatric Hematology and Oncology, Charité-Universitätsmedizin Berlin, Berlin, Germany; 6 Berlin Institute of Health, Berlin, Germany; 7 Experimental and Clinical Research Center, Max Delbrück Center for Molecular Medicine and Charité-Universitätsmedizin Berlin, Berlin, Germany; 8 Howard Hughes Medical Institute, Stanford University School of Medicine, Stanford, CA USA; 9 Present Address: Department of Neuroscience, Scripps Research, La Jolla, CA USA; 10 Present Address: Amgen Research, South San Francisco, CA USA

**Keywords:** Cancer genetics, Oncogenes, Cell division

## Abstract

Extrachromosomal DNA (ecDNA) is a prevalent and devastating form of oncogene amplification in cancer^1,2^. Circular megabase-sized ecDNAs lack centromeres, stochastically segregate during cell division^3–6^ and persist over many generations. It has been more than 40 years since ecDNAs were first observed to hitchhike on mitotic chromosomes into daughter cell nuclei, but the mechanism underlying this process remains unclear^3,7^. Here we identify a family of human genomic elements, termed retention elements, that tether episomes to mitotic chromosomes to increase ecDNA transmission to daughter cells. Using Retain-seq, a genome-scale assay that we developed, we reveal thousands of human retention elements that confer generational persistence to heterologous episomes. Retention elements comprise a select set of CpG-rich gene promoters and act additively. Live-cell imaging and chromosome conformation capture show that retention elements physically interact with mitotic chromosomes at regions that are mitotically bookmarked by transcription factors and chromatin proteins. This activity intermolecularly recapitulates promoter–enhancer interactions. Multiple retention elements are co-amplified with oncogenes on individual ecDNAs in human cancers and shape their sizes and structures. CpG-rich retention elements are focally hypomethylated. Targeted cytosine methylation abrogates retention activity and leads to ecDNA loss, which suggests that methylation-sensitive interactions modulate episomal DNA retention. These results highlight the DNA elements and regulatory logic of mitotic ecDNA retention. Amplifications of retention elements promote the maintenance of oncogenic ecDNA across generations of cancer cells, and reveal the principles of episome immortality intrinsic to the human genome.

## Abstract (web summary)

A combination of genome-wide functional screening, imaging and chromatin profiling identifies a new class of highly prevalent genomic elements that help retain extrachromosomal DNA copies in dividing cells and persist across generations.

## Main

Human cancer cells commonly amplify potent oncogenes on megabase-sized circular ecDNA molecules^8,9^ that lack centromeres and asymmetrically segragate^3–6^. This characteristic of ecDNA results in intraclonal heterogeneity in oncogene copy number and amplicon sequence and in rapid adaptations to selective pressures during cancer evolution^6,8,10–12^. During cell division, the nuclear envelope breaks down before the segregation of chromosomes, which physically attach to the mitotic spindle at centromeres and partition into daughter nuclei. Thus, the acentric nature of ecDNA raises crucial questions of how ecDNA is inherited by daughter cells and is retained in daughter nuclei after cell division. It has been well documented that viral episomes such as those of papillomaviruses, Epstein–Barr virus (EBV) and simian virus 40 tether to mitotic chromosomes to hitchhike into daughter nuclei^13–17^. Viral episome tethering is mediated by dedicated viral DNA elements, viral DNA-binding proteins and interactions with host-cell chromatin-binding proteins, such as BRD4 (refs. ^13,18,19^). Notably, ecDNA strongly colocalizes with chromosomes during mitosis^3,20–23^, which suggests that ecDNA may also tether to chromosomes during DNA segregation. However, the endogenous human DNA elements or factors that mediate this tethering process are unknown. We speculate that such DNA sequences on ecDNA would enable it to be retained in the nuclear space of dividing cancer cells, thereby serving as functional ‘retention elements’.

In principle, any ecDNA molecule that becomes amplified and persists in a cancer cell population should contain a minimum of three genetic elements: (1) a fitness element that provides an advantage to the cell when under selective pressure (for example, an oncogene or regulatory sequence); (2) origins of replication to copy itself; and (3) a retention element that promotes nuclear retention of ecDNA by mediating its segregation along with chromosomes into daughter cells during cell division. In an evolving cancer cell population, ecDNA molecules with these features would become more abundant than molecules that lack them. Although oncogenes^8,9,24^ and regulatory sequences^23,25,26^ on ecDNA and human origins of replication^27^ have been well studied, our understanding of the identity or mechanism of retention elements on human ecDNAs is limited. Here we devise a new genome-scale functional assay and apply imaging and chromatin profiling methods to elucidate the principles of genetic elements on ecDNA that promote its retention in dividing cells.

## Genetic elements drive episome retention

**Fig. 1:**
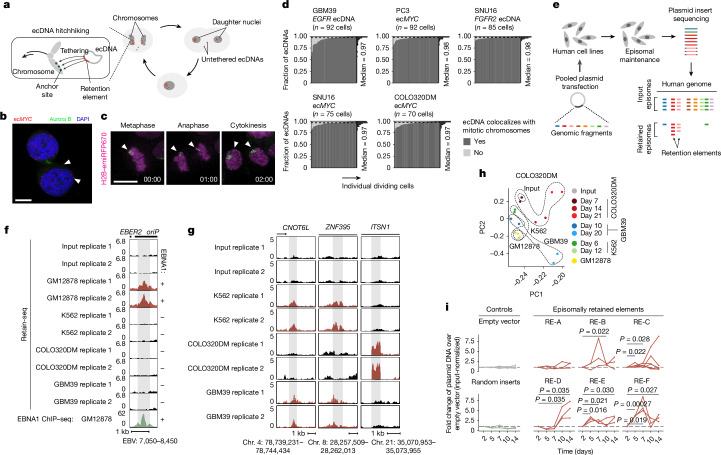
Identification of genetic elements that promote episomal DNA retention. **a**, Proposed mechanism of mitotic retention of ecDNAs in cancer cells through chromosome hitchhiking. **b**, Representative image of tethered (bottom arrowhead) and untethered (top arrowhead) ecDNA foci in mitotic PC3 cells (*n* = 92 daughter cell pairs). Scale bar, 10 µm. **c**, Representative live-cell images (*n* = 10 fields of view) showing ecDNA (labelled with TetR-mNeonGreen) colocalization with chromosomes during cancer cell division. Scale bar, 10 µm. **d**, Fractions of ecDNA with various oncogenes colocalizing with mitotic chromosomes in the following cancer cell lines: GBM39 glioblastoma cells, *EGFR* ecDNA from chromosome 7; PC3 prostate cancer cells, ec*MYC* from chromosome 8; SNU16 gastric cancer cells, ec*MYC* and *FGFR2* ecDNA from chromosome 8 and chromosome 10, respectively; COLO320DM colorectal cancer cells, ec*MYC.* Raw images were obtained from a previous publication^5^ of IF–DNA-FISH of anaphase cells. **e**, Schematic of Retain-seq. **f**, Retain-seq enrichment of a known EBV sequence that promotes viral retention. EBNA1 ChIP–seq data in EBV-transformed GM12878 cells are shown at the bottom. **g**, Retain-seq signals at three representative enriched genomic loci. Red tracks represent loci that were significantly enriched in Retain-seq screens in the corresponding cell line, thus marking these loci as retention elements in that line; black tracks indicate that the sequence was not identified as a retention element in the corresponding experiment. **h**, Principal component analysis of Retain-seq in various cell lines at different time points. **i**, Individual validation by quantitative PCR (qPCR) of six episomally retained elements (RE-A–RE-F) identified by Retain-seq experiments in the K562 cell line and amplified on COLO320DM (RE-C) and GBM39 (others) ecDNAs. Each line in the plot for a given retention element represents a single replicate. The empty vector control is the pUC19 plasmid alone, whereas the random insert control comprises the pUC19 plasmid with random insert sequences from the genome of the human GM12878 cell line. *P* values were calculated using one-sided *t*-tests.

We propose that ecDNA is retained by hitchhiking onto chromosomes during cell division through the docking of ecDNA sites, which we term retention elements, to anchor positions on chromosomes (Fig. 1a). We consider untethered ecDNAs (Fig. 1b) as lost in this context. This is because acentric DNA that fails to segregate with chromosomes is released into the cytoplasm or incorporated into micronuclei^28–30^. This DNA is subject to strong transcriptional silencing, usually not replicated or expressed and can be degraded and lost from the cell^30–32^. Live-cell time-lapse imaging of COLO320DM colorectal cancer cells with ecDNA encoding the *MYC* oncogene (ec*MYC*) showed synchronous segregation of ecDNA and chromosomal DNA during cell division (Fig. 1c). Analyses of images of DNA fluorescence in situ hybridization (FISH) paired with immunofluorescence (IF) (IF–DNA-FISH) staining of Aurora kinase B showed 97–98% colocalization of ecDNA with chromosomal DNA during segregation in multiple cell lines with ecDNA (Fig. 1d). These observations are consistent with previous reports that ecDNA synchronously segregates with chromosomes and may tether to them^3,20–23^. As these ecDNAs are derived from multiple distinct chromosomes, our results imply that functional retention elements are widely dispersed across the human genome.

**Extended Data Fig. 1:**
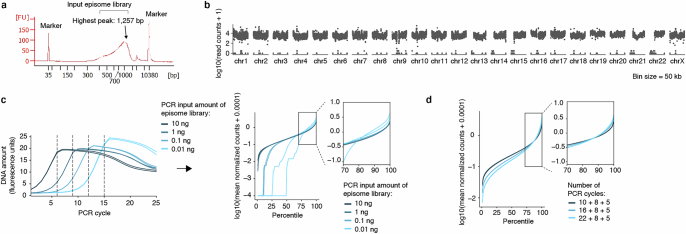
Optimization of Retain-seq library preparation. (**a**) Insert size distribution of genomic fragments included in the input mixed episome library. (**b**) Genome-wide coverage of sequenced reads derived from input episome library. (**c**) Left: Representative quantitative PCR amplification curves across varying amounts of episome library as PCR input. Right: Log-transformed mean normalized read counts of genomic bins ranked by percentile. Inset is a zoom-in of the higher-percentile genomic bins, in which a 100-fold range of DNA amounts from 0.1 ng – 10 ng of input showed highly comparable representation (despite some library dropout at 0.1 ng of input DNA) while 0.01 ng PCR input showed substantial library dropout and signs of skewing and was used to set the quality threshold for all library preparations. See Methods. (**d**) Log-transformed mean normalized read counts of genomic bins ranked by percentile. Inset is a zoom-in of the higher-percentile genomic bins showing that increasing PCR cycles during library preparation alters skewing of sequencing reads.

To broadly identify genetic sequences that may serve as retention elements on ecDNA, we developed a shotgun genetic screen, termed Retain-seq, that identifies episomally retained sequences (Fig. 1e). In brief, we created a pool of heterologous bacterial plasmids with inserts that represent random DNA sequences from the human genome (Fig. 1e and Extended Data Fig. 1a,b). We transfected the plasmid pool into multiple cell types and performed serial passaging. Retained plasmid DNA was then isolated from cells to identify enriched episomal DNA sequences through targeted sequencing of the inserts (Fig. 1e). To minimize the effects of variability in the insert size and the amount of retained plasmid DNA in the enrichment analysis due to PCR overcycling, we stopped PCR amplification at the cycle before saturation and performed all subsequent enrichment analyses by comparing the output DNA with the transfected input episomal DNA library (Extended Data Fig. 1c,d). A serial dilution experiment showed that DNA sequences with variable amounts of DNA were minimally over-represented when using this PCR strategy (Extended Data Fig. 1c). As validation for Retain-seq, we analysed levels of the *oriP* family of repeats (EBV: 7,421–8,042), the EBV genomic sequence that enables viral tethering to chromosomes mediated by the virally encoded protein EBNA1 (ref. ^33^). We observed specific episomal enrichment of *oriP* repeats only in EBNA1-positive GM12878 cells, but not in EBNA1-negative K562, COLO320DM or GBM39 cells (Fig. 1f). The Retain-seq enrichment signal coincided strongly with EBNA1 occupancy (Fig. 1f), a result consistent with the idea that EBNA1 binding to this viral element mediates episomal retention and tethering to chromosomes.

**Fig. 2:**
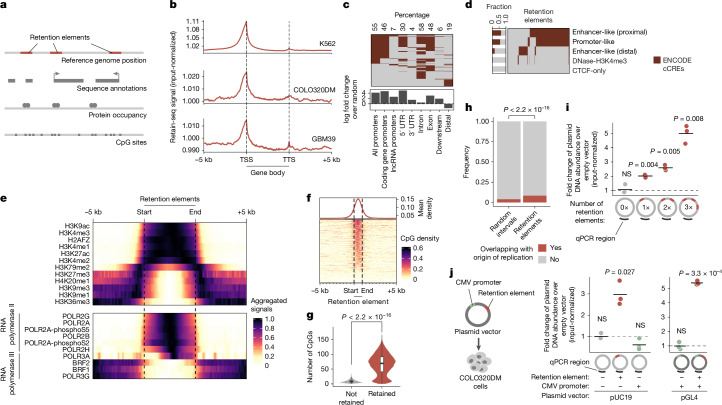
Sequence features of retention elements. **a**, Analyses of sequence features of retention elements. **b**, Input-normalized Retain-seq signals across annotated gene sequences. TTS, transcription termination site. **c**, Sequence annotations that overlap with retention elements identified in K562 cells. Percentages represent the proportion of retention elements that overlap with a given annotation class. **d**, ENCODE candidate *cis*-regulatory elements (cCREs) that overlap with retention elements identified in K562 cells. Fractions represent the proportion of retention elements that overlap with a given cCRE class. **e**, ENCODE ChIP–seq signals of the indicated histone marks and RNA polymerases II and III in K562 cells that surround retention elements identified in the same cell line. **f**, CpG density surrounding the combined set of retention elements. **g**, Number of CpG sites in genomic bins that overlap with retention elements (*n* = 18,494) compared with those that do not (*n* = 2,543,727). Box centre, line median; limits, upper and lower quartiles; whiskers, 1.5× the interquartile range. **h**, Fraction of origins of replication (identified by SNS-seq in K562 cells) that overlap with retention elements identified in K562 cells and random genomic intervals. **i**, Retention of plasmids that contain one, two or three copies of a retention element (RE-C; red segments in schematic) in COLO320DM cells, analysed by qPCR. Fold changes were computed using plasmid levels at day 14 after transfection, normalizing to levels at day 2 to adjust for different transfection efficiencies across conditions (three biological replicates). **j**, Left, schematic of transfection of plasmids with a CMV promoter and/or a retention element (RE-C) into COLO320DM cells. Right, retention of plasmids that contain a CMV promoter and/or a retention element in COLO320DM cells, assessed by qPCR (three biological replicates). Data for two different plasmid backbones, pUC19 and pGL4, are shown. *P *values were computed using two-sided Wilcoxon rank-sum tests (**g**), one-sided hypergeometric tests (**h**) or one-sided *t*-tests (**i**,**j**). NS, not significant.

**Extended Data Fig. 2:**
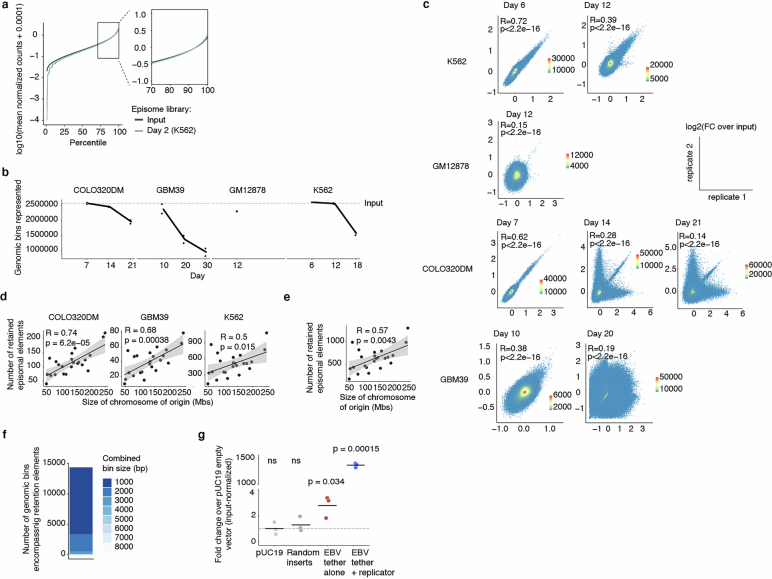
Distribution of Retain-seq reads across the genome and experimental replicates. (**a**) Log-transformed mean normalized read counts of genomic bins ranked by percentile. Inset is a zoom-in of higher-percentile genomic bins showing that transfection, represented by the day 2 episome library, results in minimal dropout that does not substantially skew the sequence representation compared to the input episomal library. (**b**) Loss of genome-wide representation in episomal insert sequences relative to the input library over time in four cell lines assayed with Retain-seq. (**c**) Correlations between experimental replicates of Retain-seq across time points from different cell lines. (**d**) Correlation (Pearson’s *R*; error bands represent 95% confidence intervals) between the numbers of episomally retained elements and the sizes of their chromosomes of origin in experiments performed in various cell lines. (**e**) Correlation (Pearson’s *R*; error bands represent 95% confidence intervals) between the numbers of episomally retained elements and the sizes of their chromosomes of origin across all cell lines. (**f**) Distribution of genomic bin sizes containing retention elements (median 1 kb; s.d. 0.604 kb). (**g**) Retention of plasmids containing random genomic inserts, the EBV tethering sequence alone, or the entire EBV origin (containing both tethering and replicative sequences) compared to pUC19 in GM12878 cells (three biological replicates). Fold changes were computed using plasmid levels at day 14 post-transfection, normalizing to levels at day 2 to adjust for differential transfection efficiency across conditions. *P*-values computed by one-sided t-test.

**Extended Data Fig. 3:**
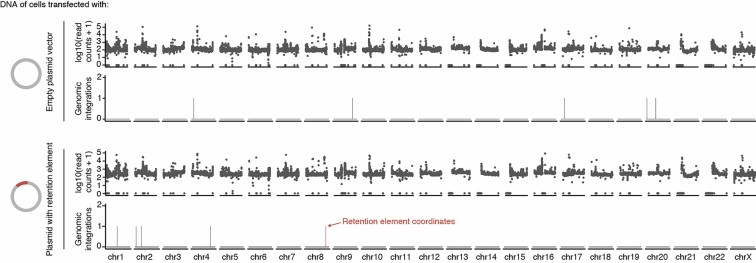
Chromosomal integration events of transfected plasmids containing a retention element are stochastic and occur at near-background levels. Genome-wide read coverage (non-overlapping 50 kb bins) and detection of chromosomal integration events (events per bin) of transfected plasmids in single-molecule long-read nanopore sequencing from cells transfected with either an empty plasmid vector (pUC19; top) or plasmid containing a retention element (pUC19_RE-C; bottom).

Next, we analysed retained episomal DNA from multiple time points across two ecDNA-positive cell lines, COLO320DM and GBM39, and one ecDNA-negative cell line, K562 (Fig. 1g). The sequence representation of the transfected library was comparable to that of the input episomal library; thus, the latter was used in subsequent analyses for identifying enriched elements (Extended Data Fig. 2a). We then filtered out time points at which genome representation of the episomes dropped below our data-quality threshold using the serial dilution experiment (Methods and Extended Data Fig. 2b). Owing to variations in transfection efficiencies and growth rates across cell lines, we observed different levels of stochastic drift in the retained episomal library between replicates over time (Fig. 1h and Extended Data Fig. 2c). To first capture retention elements with potential activity in any cell line, we identified a combined set of 14,353 retention elements (Extended Data Fig. 2d,e). Most retention elements were captured in 1-kb genomic segments (Extended Data Fig. 2f). To validate the ability of retention elements to retain episomal DNA in cells, we individually cloned six retention elements originally identified in the Retain-seq experiment in K562 cells into the pUC19 plasmid backbone and transfected these plasmids individually into K562 cells. These particular retention elements were chosen for validation because they also overlapped with the coordinates of ecDNAs found in COLO320DM cells and in GBM39 cells. Five out of the six plasmids with retention elements were retained in K562 cells at higher levels than in both the empty vector control and plasmids with random sequence inserts. This result validates the activity of retention elements identified by Retain-seq (Fig. 1i). Although a subset of retention elements was both enriched and individually validated in multiple cell types (for example, RE-C; Figs. 1i and 2j), most seemed to be unique to each cell type, which might reflect cell-type specificity or technical variation across cell lines. A positive-control plasmid with the EBV-tethering sequence alone displayed an increase in plasmid persistence of comparable magnitude relative to an empty vector control (Extended Data Fig. 2g). This result shows that retention elements identified in the human genome promote episomal DNA retention to similar extents as known viral sequences. A retention element does not increase genomic integration of plasmids (Extended Data Fig. 3), which rules out preferential integration of episomal elements into chromosomes as a mechanism of retention. Together, these results suggest that episomal retention elements are broadly distributed across the human genome.

## Retention elements comprise active DNA

**Extended Data Fig. 4:**
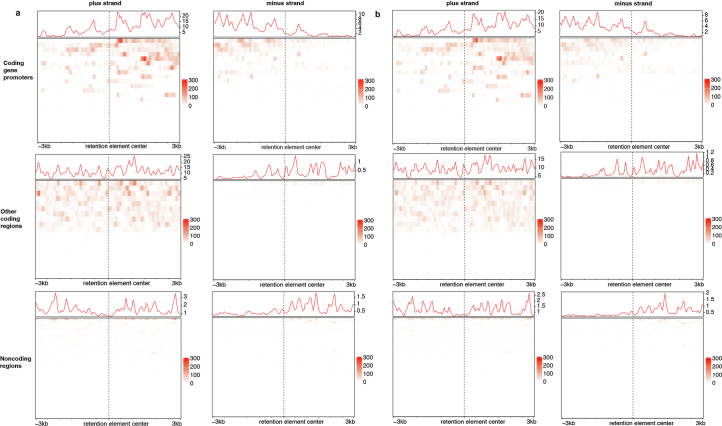
Many, but not all retention elements represent sites of active nascent transcription. (**a**) Histograms and heatmaps of COLO320DM GRO-seq signal from biological replicate 1, computed over 50 bp bins within 3 kb of the midpoints of retention elements located within the genomic coordinates of the COLO320DM ecDNA. Retention elements were divided into 3 categories based on overlap with genomic annotations: those that overlap with coding gene promoters, other portions of coding genes, or noncoding regions. X-axis directionality is consistent for both strands. (**b**) Heatmap of COLO320DM GRO-seq signal from biological replicate 2 within 3 kb of the midpoints of retention elements located within the genomic coordinates of the COLO320DM ecDNA.

**Extended Data Fig. 5:**
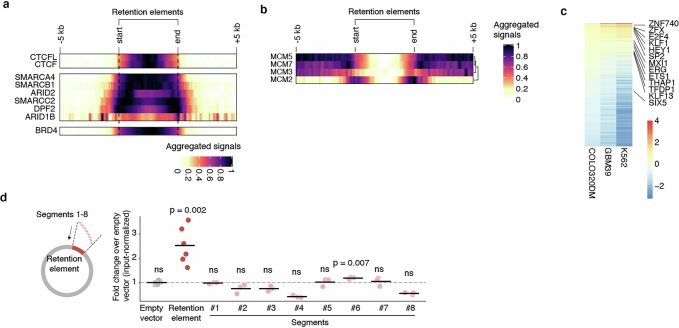
Additional sequence features of retention elements. (**a**) ENCODE ChIP-seq signals of the indicated proteins in K562 cells surrounding retention elements identified in the same cell line. (**b**) ENCODE ChIP-seq signals of components of the replication licensing complex in K562 cells surrounding retention elements identified in the same cell line. (**c**) Motif enrichment (log2 fold change) of transcription factor motifs in retention element intervals identified in COLO320DM, GBM39, and K562 cells relative to random genomic intervals. (**d**) Episomal retention of plasmids containing 8 overlapping 500-bp tiles of a retention element (RE-C) in COLO320DM cells measured by quantitative PCR (six biological replicates for empty vector and retention element conditions, three for others). *P*-values computed by one-sided t-test.

We next sought to characterize the sequence features of retention elements (Fig. 2a). Retention elements were highly enriched at transcription start sites (TSSs) and in 5′ untranslated regions (UTRs) of genes (Fig. 2b,c). By contrast, retention elements were depleted across the large stretches of distal intergenic regions (Fig. 2c). Retention elements were broadly associated with regions of active chromatin, showing strong enrichment at gene promoters and enhancers (Fig. 2c,d) and at sites occupied by both actively elongating and paused RNA polymerase II (Fig. 2e). As expected, owing to their overlap with promoter sequences, a substantial proportion of retention elements represented sites of nascent transcription (Extended Data Fig. 4a,b). However, the presence of retention elements that are not actively transcribed and the fact that most ecDNAs are maintained in the nucleus even after transcription inhibition by triptolide treatment^6^ suggest that transcription may not be necessary for the function of all retention elements (Extended Data Fig. 4a,b). Retention elements were also preferentially bound by the SWI/SNF chromatin remodelling complex, BRD4, CTCF and histones with active marks such as H3K27ac, H3K4me3 and H3K9ac (Fig. 2e and Extended Data Fig. 5a). Notably, retention elements showed an absence of overlap with RNA polymerase III or repressive histone marks such as H3K9me3 and H3K27me3 (Fig. 2e). CpG density was also increased in retention elements (Fig. 2f,g), a finding consistent with the idea that regions of active chromatin in the genome typically contain CpG-dense DNA sequences^34^. Because retention elements are CpG-rich and do not seem to be heterochromatinized, they probably represent a separate class of sequences from AT-rich scaffold matrix attachment regions^35^ and rely on diverse protein factors for function. Notably, we observed only minor overlap (about 8%) of retention elements with origins of replication and low occupancy of replication licensing complexes (MCM2–MCM7) at retention elements. This result suggests that retention elements do not promote episomal DNA enrichment by serving as origins of replication (Fig. 2h and Extended Data Fig. 5b). Furthermore, transfection with plasmids with either validated retention elements or a known EBV-tethering sequence showed similar levels of retention in cells. By contrast, incorporation of the full EBV origin, including a replicator sequence, markedly increased plasmid DNA content by two orders of magnitude. This finding supports the conclusion that retention elements alone do not broadly induce DNA replication (Extended Data Fig. 2g).

Episomal retention increased with the number of retention elements (Fig. 2i). This additive effect also suggests that retention elements are functionally distinct from centromeres, as the presence of more than one centromere per episome or chromosome leads to opposing kinetochores pulling on the same DNA, which leads to DNA fragmentation and loss^36^. Notably, although we observed enrichment of gene promoters in retention elements (Fig. 2b–d), the constitutive cytomegalovirus (CMV) promoter did not promote episomal retention alone (Fig. 2j). This observation shows that an active promoter itself is not sufficient to enable DNA retention and suggests that additional sequence-specific interactions may be required. Consistent with this idea, similar DNA motifs of chromatin-binding proteins were enriched across retention elements identified in multiple cell lines. This result suggests that sequence features of retention elements may converge despite variations in the enriched intervals themselves across cell lines (Extended Data Fig. 5c). As a preliminary effort to identify a minimal sequence sufficient for episomal retention, we split a retention element into eight overlapping tiles and individually assayed each segment (Extended Data Fig. 5d). However, no individual segment enabled episomal retention to the extent of the original larger sequence, which indicates a possible reliance on combinatorial interactions across multiple sites in this element (Extended Data Fig. 5d). Together, these results show that retention elements are pervasive, additive and functionally composite DNA elements.

## Retention elements tether to chromosomes

**Fig. 3:**
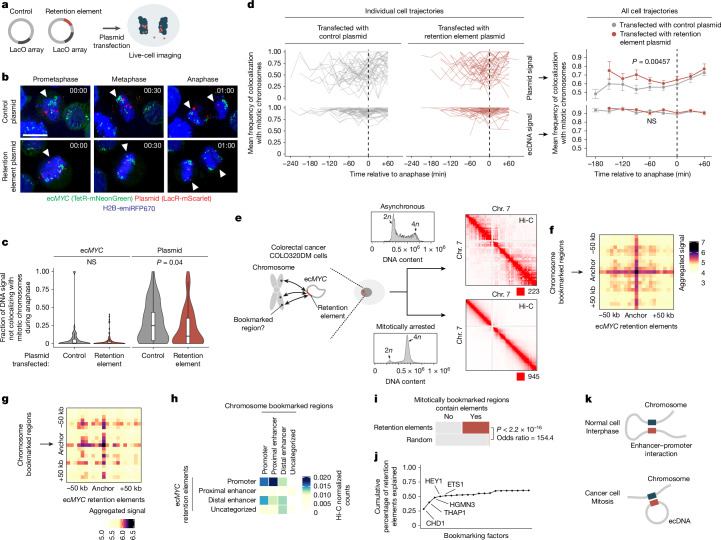
Retention elements promote extrachromosomal interactions with chromosomes during mitosis. **a**, Schematic of the live-cell imaging experiment. **b**, Representative live-cell time-lapse images of dividing COLO320DM cells with labelled ec*MYC* after transfection with a plasmid containing a retention element or an empty vector control. Scale bar, 10 µm. **c**, Fraction of DNA signals not colocalizing with mitotic chromosomes during anaphase. *n* = 51 (control), *n* = 83 cells (retention element). Box plot parameters are as described in Fig. 2. **d**, Individual (left) and mean (right) cell trajectories of DNA signal colocalization with chromosomes throughout mitosis. *n* = 42 (control), *n* = 45 (retention element) cells. Mean cell trajectories include all time points with >3 cells. Error bars show the s.e.m. Vertical dashed lines indicate anaphase. **e**, Hi-C interaction maps in asynchronous or mitotically arrested COLO320DM cells. Numbers at bottom right below far right plots indicate maximum count values in corresponding color scales. Density plots show flow cytometry analyses of DNA content. **f**,**g**, APA of Hi-C data of asynchronous (**f**) and mitotically arrested (**g**) COLO320DM cells. Heatmaps are summed percentile matrices of pairwise interactions between chromosome bookmarked regions and a combined set of ec*MYC* retention elements with 5-kb resolution. **h**, Hi-C heatmap of pairwise interactions in mitotically arrested COLO320DM cells between ec*MYC* retention elements and chromosome bookmarked regions with ENCODE cCRE annotations. **i**, Mitotically bookmarked regions that overlap with retention elements or matched-size random genomic intervals. **j**, Cumulative distribution of retention elements that contain binding sites of bookmarking factors, ordered by factor enrichment relative to random genomic intervals. **k**, ecDNA**–**chromosome interactions recapitulate enhancer–promoter interactions. Gene expression in interphase cells is activated by an interaction between enhancer (blue) and promoter (red) sequences on the same chromosome. We propose that ecDNA retention in mitotic cells is mediated by an analogous intermolecular contact between promoter-like retention elements (red) on ecDNA and enhancer-like, or less commonly, promoter-like bookmarked sites (blue) on the chromosome.* P* values were calculated using two-sided Wilcoxon rank-sum tests (**c**), two-sided paired *t*-tests (**d**) or two-sided Fisher’s exact tests (**i**).

**Extended Data Fig. 6:**
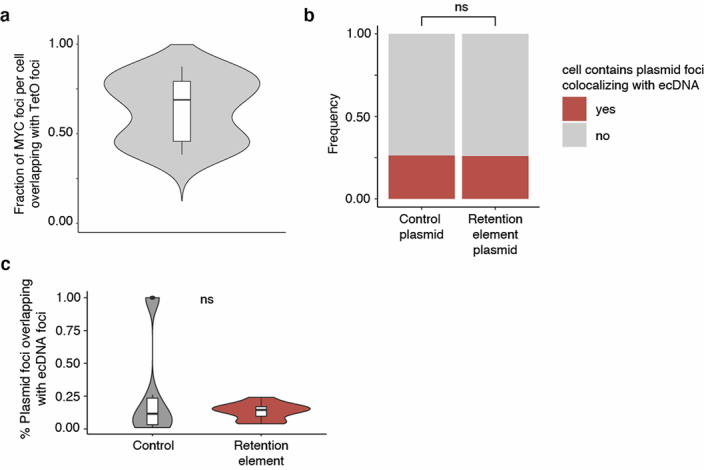
Summary of COLO320DM live cell imaging line. (**a**) Fraction of *MYC* ecDNA foci with overlapping TetO foci for each metaphase cell, indicating the percentage of labeled ecDNAs per cell (*n* = 20 cells). Box plot parameters as in Fig. 2. (**b**) Frequency of cells containing plasmid foci (either control or retention element plasmids) that colocalize with TetO-labeled ecDNA foci. *n* = 38 (control) and *n* = 46 (retention element) cells. *P*-value determined by one-sided hypergeometric test. (**c**) Percentages of plasmid foci area (either control or retention element plasmids) that colocalize with TetO-labeled ecDNA foci. *n* = 10 (control) and *n* = 12 (retention element) cells; only the subset of cells with plasmid foci that at least partially overlap with ecDNA foci are plotted here. Box plot parameters as in Fig. 2. *P-*value computed using a two-sample Wilcoxon test.

Next, we asked whether retention elements enable episomal DNA to tether to chromosomes during DNA segregation. Using the COLO320DM cell line with ec*MYC* edited to contain a Tet-operator (TetO) array, we introduced plasmid DNA containing a Lac-operator (LacO) array. We then assessed the localization of the plasmid and ecDNA during DNA segregation using fluorescence labelling and live-cell imaging (Fig. 3a,b and Extended Data Fig. 6a). Plasmids with a retention element displayed significantly increased colocalization with chromosomes throughout mitosis compared with the empty vector control (Fig. 3c,d). A single retention element more than halved the probability of failure of chromosome hitchhiking of the linked episome from 25% to 10.4% per mitotic event (Fig. 3c). This difference was not observed in the TetO ecDNA signals between the two plasmid transfection conditions, a result that validated the uniform analysis across conditions (Fig. 3c,d). This observation supports the idea that retention elements may increase episomal DNA retention by promoting its tethering to mitotic chromosomes. Ectopic plasmids with a retention element did not necessarily colocalize with endogenous ecDNAs (Fig. 3b and Extended Data Fig. 6b,c), which indicates that retention elements confer autonomous retention activity.

## Episomal contact with mitotic bookmarks

**Extended Data Fig. 7:**
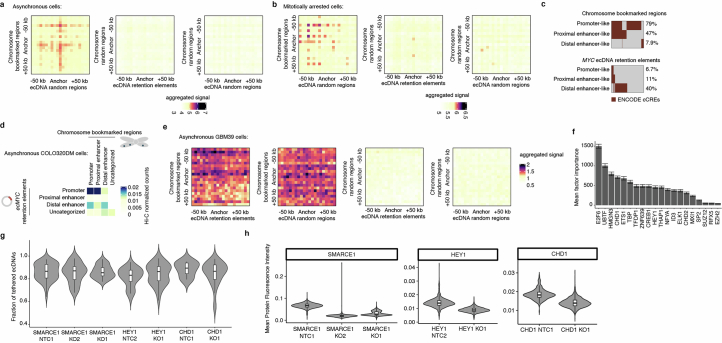
Chromatin interactions and functional annotations of chromosome bookmarked regions and *ecMYC* retention elements. (**a-b**) Aggregated peak analysis (APA) of Hi-C data of asynchronous (a) and mitotically arrested (b) COLO320DM cells. Heatmaps are summed percentile matrices of pairwise interactions between previously reported chromosome bookmarked regions (Methods) and a combined set of retention elements identified on the *MYC* ecDNA with 5-kb resolution, in which the chromosome bookmarked regions and/or the *ecMYC* retention elements are randomized. (**c**) Chromosome bookmarked regions or *ecMYC* retention elements with the indicated ENCODE cCRE annotations. (**d**) Hi-C heatmap of pairwise interactions between the *MYC* ecDNA retention elements and chromosome bookmarked regions with the indicated ENCODE cCRE annotations in asynchronous cells. Hi-C counts are normalized to number of interactions as well as bin sizes. (**e**) APA of Hi-C data of asynchronous GBM39 cells. (**f**) Importance scores (error bars show s.e.m.) indicating the relative contribution of each bookmarking factor to the cumulative distribution of retention elements. Scores represent the mean incremental number of retention elements containing binding sites for each factor over 1000 randomized cumulative distributions of the 20 bookmarking factors shown. Bookmarking factors are displayed in order of ChIP-seq peak enrichment within retention elements relative to random genomic intervals. (**g**) Fraction of tethered ecDNAs following CRISPR/Cas9 knockouts of selected bookmarking factors in mitotic COLO320DM cells. Box plot parameters as in Fig. 2. *n* = 55 (SMARCE1 NTC1), *n* = 42 (SMARCE1 KO1), *n* = 39 (SMARCE1 KO2), *n* = 34 (HEY1 NTC2), *n* = 33 (HEY1 KO1), *n* = 8 (CHD1 NTC1), *n* = 36 (CHD1 KO1) cells. (**h**) Mean immunofluorescence intensity of selected bookmarking factors in cells receiving targeting guide RNAs or non-targeting control (NTC) guides. *n* = 1874 (SMARCE1 NTC1), *n* = 2217 (SMARCE1 KO1), *n* = 1371 (SMARCE1 KO2), *n* = 1459 (HEY1 NTC2), *n* = 1976 (HEY1 KO1), *n* = 316 (CHD1 NTC1), *n* = 2730 (CHD1 KO1) cells. Box plot parameters as in Fig. 2.

Our live-cell imaging analysis showed that a retention element promotes the tethering of plasmids to chromosomes during mitosis. Therefore, we asked whether retention elements on oncogene-containing ecDNAs in cancer cells (that is, genomic intervals in the ecDNA that coincide with retention element intervals identified by Retain-seq) contact specific sites on chromosomes. Although chromosomes are compacted 10,000-fold during mitosis, some genomic sites remain accessible and are stably bound by transcription factors throughout mitosis^37–43^, a phenomenon termed mitotic bookmarking. To first interrogate whether ecDNA–chromosome interactions occur at mitotically bookmarked loci, we performed genome-wide chromosome conformation capture using Hi-C on mitotically arrested COLO320DM cells to analyse pairwise DNA interactions between ec*MYC* and chromosomes (Fig. 3e). As expected, pairwise chromatin interaction maps showed plaid patterns of long-range interactions in asynchronous cells. By contrast, mitotically arrested cells showed substantial loss of these long-range interactions owing to chromatin condensation (Fig. 3e), a result consistent with results from previous Hi-C studies^44^. Next, we performed aggregate peak analysis (APA) to measure enrichment of Hi-C signals in pairs of loci, with one partner on ec*MYC* containing a retention element and the other partner on a chromosome containing a mitotically bookmarked region (Fig. 3f,g). We observed enrichment of Hi-C contacts between chromosome bookmarked regions and ec*MYC* retention elements in asynchronous cells. These elements were retained in the condensed chromatin of mitotically arrested cells despite increased background noise (Fig. 3f,g). By contrast, we did not observe focal interactions when either or both the chromosomal or extrachromosomal regions were randomized (Extended Data Fig. 7a,b). These data suggest that focal interactions occur between retention elements on ecDNA and mitotically bookmarked regions on chromosomes both in interphase and during mitosis. This behaviour is analogous to that of the EBV episomal genome, which also remains associated with chromosomes throughout the cell cycle^33^. The majority of chromosome bookmarked regions overlapped with promoters or proximal enhancer-like elements, whereas ec*MYC* retention elements consisted of distal enhancer-like elements and promoters (Extended Data Fig. 7c). Notably, retention elements on ec*MYC* that overlapped with promoters showed increased Hi-C contact with proximal enhancer-like elements and promoters at chromosome bookmarked regions. Conversely, retention elements on ec*MYC* that overlapped with distal enhancer-like elements showed increased Hi-C contact with chromosome bookmarked loci that originated from promoters (Fig. 3h and Extended Data Fig. 7d). We also performed APA on Hi-C data from asynchronous GBM39 cells. However, results of this analysis were inconclusive, probably because of the small sampling size. That is, ecDNAs in this cell line contain a low number of retention elements (Extended Data Fig. 7e).

Because factors that promote ecDNA retention through chromosomal hitchhiking should bind to condensed chromosomes during mitosis, mitotic bookmarking factors are plausible candidates as mediators of ecDNA retention. Nearly half of the mitotically bookmarked regions were also identified as retention elements, which were highly enriched compared with randomly selected genomic intervals of the same size (Fig. 3i). Many putative bookmarking factors represented by ChIP–seq data in K562 cells (obtained from the ENCODE consortium^45^) showed occupancy in retention elements, with as few as five bookmarking factors cumulatively binding >50% of retention element intervals (Fig. 3j). Notably, a subset of bookmarking factors consistently bound more retention elements than others, which indicated that some factors may disproportionately contribute to retention element activity (Extended Data Fig. 7f). However, individual CRISPR-mediated knockout of three enriched bookmarking factors did not result in widespread untethering of ecDNA in mitotic COLO320DM cells. This result suggests that mitotic ecDNA retention involves complexes of multiple redundant DNA-binding proteins on active chromatin^46^ (Extended Data Fig. 7g,h). Together, these observations support the idea that ecDNA–chromosome interactions in mitotic cancer cells intermolecularly recapitulate promoter–enhancer interactions (Fig. 3k).

## Cancer ecDNAs contain retention elements

**Fig. 4:**
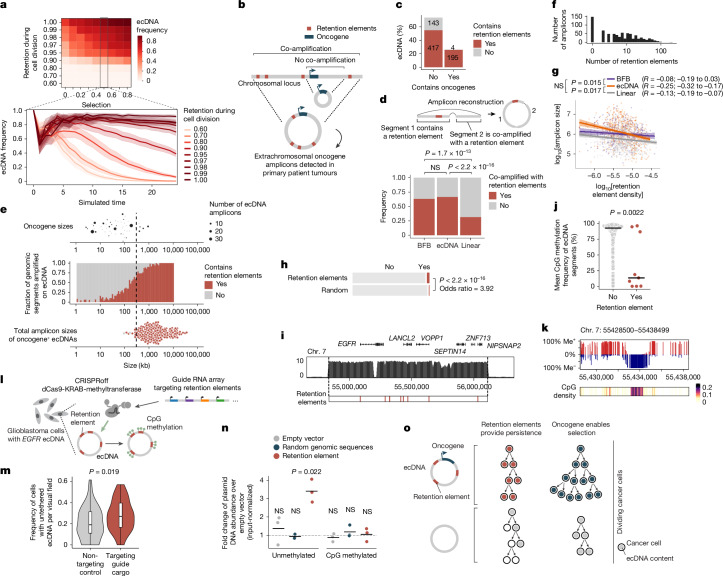
Retention elements enable selection of oncogene-containing ecDNAs in cancer. **a**, Mean frequency (>10 independent replicates) of cells with ≥1 ecDNA in simulations. Shaded area, s.e.m. **b**, Analysis of retention element co-amplification with oncogenes on ecDNA in patient tumours. **c**, ecDNA amplicons that contain retention elements and/or oncogenes. **d**, Top, schematic of an ecDNA segment without retention elements co-amplified with a retention element. Bottom, frequency of co-amplification with retention elements in BFB, ecDNA or linear amplicons for genomic segments without retention elements. **e**, Top to bottom, oncogene sizes on ecDNA, frequency of genomic segments that contain retention elements sorted by size, and total ecDNA amplicon sizes. **f**, Schematic of experiment to analyse the distribution of retention element numbers among ecDNAs. **g**, Correlation (Pearson’s *R* with 95% confidence intervals) between local density of retention elements (Methods) and amplicon size. The plot shows the linear fit using ordinary least squares with 95% confidence intervals. **h**, Circular microDNAs in five human cell lines that overlap with retention elements or matched-sized random genomic intervals detected using circle-seq. **i**, Increased WGS coverage of *EGFR* ecDNA in GBM39 cells and retention element positions. **j**, 5mC CpG methylation of retention elements (*n* = 9 segments) compared with matched-sized sequence intervals (*n* = 1,235 segments) in GBM39 ecDNA. **k**, 5mC methylation (Me^+^ or Me^–^) and density of CpG sites surrounding a retention element on GBM39 ecDNA. **l**, Site-specific methylation of retention elements by CRISPRoff. **m**, Frequency of GBM39 cells that contain untethered ecDNA foci 5 days after transfection. *n* = 60 (nontargeting) and *n* = 50 (targeting) visual fields. Box plot parameters are as described in Fig. 2. **n**, Plasmid retention after methylation in COLO320DM cells, as assessed by qPCR (three biological replicates). **o**, Retention elements and oncogenes on ecDNA (left) confer retention and selection, respectively, two processes that shape the evolution of cancer cell lineages (right). *P*  values were calculated using one-sided tests of equal proportions (**d**), two-sided Fisher’s *z*-tests (**g**), two-sided Fisher’s exact tests (**h**), two-sided Wilcoxon rank-sum tests (**j**), two-sided Mann–Whitney–Wilcoxon tests (**m**) or one-sided *t*-tests (**n**).

**Extended Data Fig. 8:**
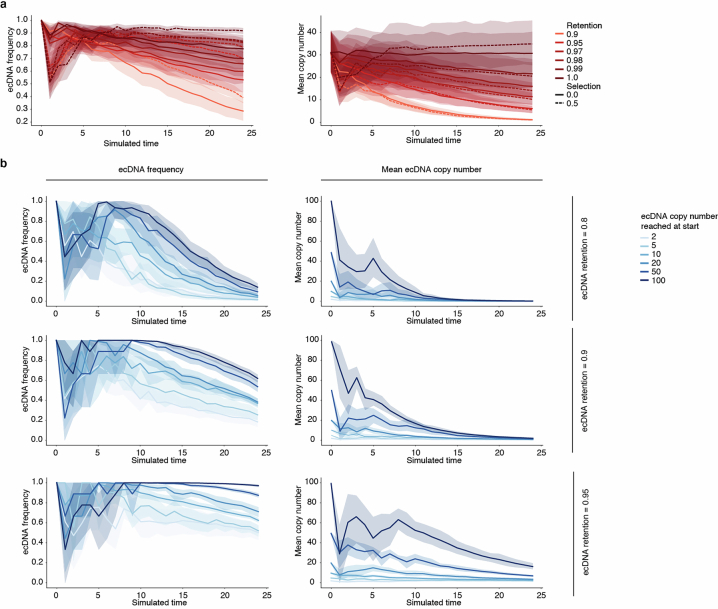
Evolutionary modeling of ecDNA retention and selection in growing cancer cell populations. (**a**) Time-resolved simulated trajectories of ecDNA frequency and mean copy number (95% confidence intervals shaded) across 25 simulated time units with various selection and retention values. (**b**) Time-resolved simulated trajectories of ecDNA frequency and mean copy number (95% confidence intervals shaded) across 25 simulated time units stratified by the number of initial ecDNA copies present in the parental cell. Trajectories are reported for various levels of retention. Selection is fixed at 0.5.

Although retention elements promote the maintenance of episomal DNA in dividing cells, ecDNAs also provide selective advantages to cancer cells by encoding oncogenes. Thus, ecDNAs can theoretically become amplified in a cell population owing to selection despite imperfect retention during cell division. To explore the relative contributions of retention and selection on ecDNA amplification, we simulated growing cancer cell populations by adapting an evolutionary framework^6^ to model imperfect retention. ecDNAs were amplified with increased selection as expected; however, they were rapidly lost when the retention fidelity of ecDNAs per cell division dropped below 0.9 (Fig. 4a and Extended Data Fig. 8a). This result suggests that a markedly high level of mitotic retention is a prerequisite for selection to drive ecDNA amplification. Notably, this minimum predicted level matched the experimentally observed mitotic retention rate (10% failure rate per mitosis) conferred by a single retention element, on the basis of live-cell imaging (Fig. 3c). Mitotic retention remained important even after ecDNAs reached high copy numbers. That is, imperfect retention led to loss of ecDNAs over time, even in cells that had already reached high copy numbers and were under positive selection (Extended Data Fig. 8b).

**Extended Data Fig. 9:**
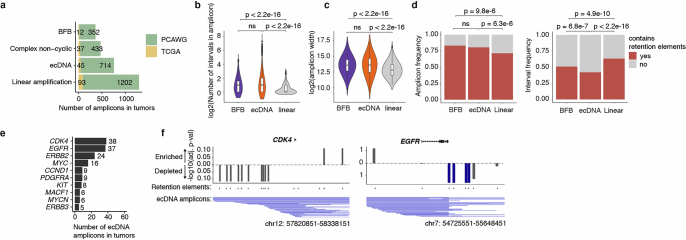
Summary statistics of DNA amplifications identified in WGS data of patient tumor samples. (**a**) Patient samples analyzed and classification of amplicons identified. (**b**) Number of genomic intervals implicated in each amplicon (i.e., degree of genomic rearrangement within an amplicon) across amplicon classes. *n* = 364 (BFB), *n* = 759 (ecDNA), and *n* = 1295 (linear) amplicons. Box plot parameters as in Fig. 2. *P*-values computed using two-sample Wilcoxon tests. (**c**) Amplicon widths (in bp) across amplicon classes. *n* = 364 (BFB), *n* = 759 (ecDNA), and *n* = 1295 (linear) amplicons. Box plot parameters as in Fig. 2. *P*-values computed using two-sample Wilcoxon tests. (**d**) Frequency of amplicons (left) or amplicon intervals (segments; right) containing at least one retention element across classes. *P-*values determined by one-sided hypergeometric test. (**e**) Top 10 oncogenes most frequently amplified as ecDNAs in analyzed patient samples. (**f**) Frequency of co-amplification of *CDK4* (left) or *EGFR* (right) with neighboring retention elements (within 250 kb of gene midpoint) in observed ecDNA amplicons (below each plot) reconstructed from patient samples relative to corresponding oncogene-containing random genomic intervals drawn from an equivalent size distribution.

We next asked whether copy-number amplified, oncogene-containing ecDNAs from patient tumour samples contain retention elements (Fig. 4b). Analyses of focal amplifications in whole-genome sequencing (WGS) data from two patient cohorts (Extended Data Fig. 9a) revealed that nearly all oncogene-containing ecDNAs have retention elements (98%; Fig. 4c). DNA segments that did not contain retention elements were often connected with those containing retention elements on ecDNAs but not chromosomal linear amplicons, even after adjusting for rearrangement events (Fig. 4d and Extended Data Fig. 9b). Breakage–fusion–bridge (BFB) amplifications, which can generate both ecDNAs and complex linear amplicons, also showed similar enrichment of retention element co-amplification (Fig. 4d). Moreover, observed ecDNAs were around tenfold larger in size (>1 Mb) than the oncogene-coding sequences and their cognate regulatory elements (around 100 kb). Thus, nearly all observed ecDNA sequence coordinates encompass large segments of additional DNA sequence to reach megabase-scale sizes. At these lengths, the ecDNAs were highly likely to contain multiple retention elements (Fig. 4e,f), which serially increase the likelihood of extrachromosomal maintenance (Fig. 2i). By contrast, linear amplicons covered a more dispersed range of sizes, thereby frequently containing smaller amplicons that were less likely to have retention elements (Extended Data Fig. 9c,d).

To address whether the distribution of retention elements near an oncogene shapes amplification of the DNA sequence, we analysed the degree of co-amplification between each specific retention element and each of two oncogenes frequently amplified on ecDNA: *EGFR* and *CDK4* (Extended Data Fig. 9e). We observed skewing of ecDNA amplicon distributions in the noncoding regions that contained retention elements upstream of the oncogene promoters (Extended Data Fig. 9f). Selection for large amplicons may be due to either inclusion of retention elements or co-amplification of distal enhancers^25^. However, examination of the distributions of retention elements across all ecDNA loci showed that the amplicon size decreased as the local density of retention elements increased (Fig. 4g). This result suggests that regions of the genome that are sparsely populated with retention elements are selected with larger ecDNA sequences that are more likely to capture retention elements. Conversely, smaller ecDNA sequences are selected in regions that are densely populated with retention elements. This relationship was observed to a significantly greater extent in ecDNAs than in linear amplicons (Fig. 4g) across a broad range of cancer types expressing various oncogenes. These results support the premise that co-amplification of multiple retention elements with oncogenes on ecDNAs provides a selective advantage and shapes ecDNA structure.

Although large clonally selected ecDNAs are frequently observed in cancer, small (sub-kilobase-sized) nonclonal extrachromosomal circular DNAs (eccDNAs, also termed microDNAs) that often lack gene-encoding sequences have been detected in healthy somatic tissues^47,48^. These microDNAs are not maintained at amplified copy numbers and result from DNA fragmentation from across the entire genome^47^. The majority (96.5%) of microDNAs lack retention elements, as expected; nonetheless, we observed an enrichment of retention elements in observed microDNA sequences in LNCaP, C4-2, PC-3, OVCAR8 and ES-2 cell lines compared to random^49^. This finding is consistent with the idea that ecDNA that contains retention elements may be more persistent in cells (Fig. 4h). Collectively, these results show that the distribution of retention elements in the genome shapes the presence and sequence of DNA outside chromosomes.

## Methylation silences retention elements

**Extended Data Fig. 10:**
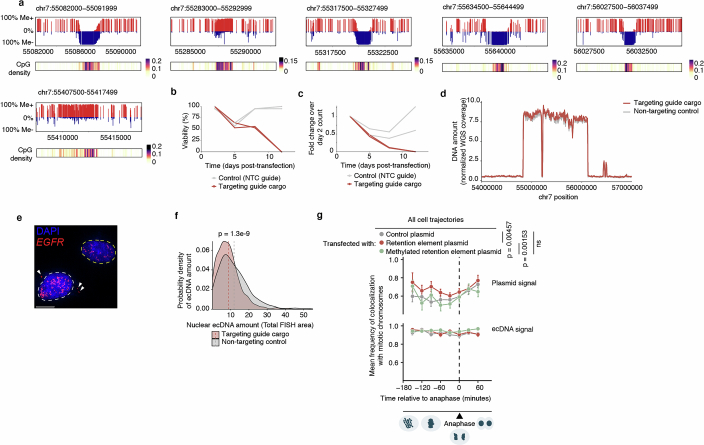
Hypomethylated CpG state is essential to retention element function. (**a**) 5mC methylation status of individual CpG sites and their density within and surrounding retention elements on the *EGFR* ecDNA in GBM39 cells as measured in single-molecule long-read nanopore sequencing. (**b**) Viability of cells expressing CRISPRoff and a targeting guide cargo or non-targeting control over time. Cells were sorted at day 2 post-transfection and tracked until day 12, when no live targeted cells remained. Each line represents an independent biological replicate. (**c**) Counts of cells expressing CRISPRoff and a targeting guide cargo or non-targeting control guide RNA over time. Cells were sorted at day 2 post-transfection and tracked until day 12, when no live targeted cells remained. Each line represents an independent biological replicate. (**d**) Abundance of ecDNA following CpG methylation of retention elements by CRISPRoff at 5 days post-transfection compared to cells expressing a non-targeting control guide RNA in WGS coverage. (**e**) Representative image showing ecDNA foci lost from the nucleus in an interphase GBM39 cell 5 days after transfection with CRISPRoff and a guide cargo targeting retention elements (*n* = 50 image positions). Scale bar, 10 µm. (**f**) Abundance of nuclear ecDNA measured by nuclear *EGFR* DNA FISH signal at 5 days after transfection of CRISPRoff and guide cargo targeting retention elements compared to cells expressing a non-targeting control guide RNA*. P*-value computed using two-sided two-sample Kolmogorov-Smirnov test. (**g**) Mean cell trajectories of methylated retention element plasmid (*n* = 51 cells) or *ecMYC* DNA signal colocalization with chromosomes throughout mitosis. Mean cell trajectories include all time points with more than 3 cells. Measurements for the control and unmethylated retention element plasmid conditions are reproduced from Fig. 3d. Error bars show s.e.m. *P*-values determined by two-sided paired t-test of the means.

Retention elements are CpG-rich promoters and associate with chromosomal bookmarked regulatory elements. Therefore, we speculated that cytosine methylation of these CpG sites, which are known to silence promoter activity and inhibit transcription factor binding^50^, may affect interactions between retention elements and cellular components that promote their retention. Retention elements on ecDNA were hypomethylated (Fig. 4i–k). Six out of the nine candidate retention-element intervals in *EGFR* ecDNA in GBM39 glioblastoma neurospheres were significantly demethylated compared with all other sequence intervals of 1-kb width on the same ecDNA (Fig. 4j). Analyses of *EGFR* ecDNA in GBM39 cells by single-molecule long-read sequencing^12^ confirmed specific and focal hypomethylation at retention elements (Fig. 4j,k and Extended Data Fig. 10a). To test whether CpG methylation affects ecDNA retention, we used a catalytically dead Cas9 fused to DNA methyltransferase (CRISPRoff)^51^ to program site-specific CpG methylation simultaneously on five hypomethylated retention elements on *EGFR* ecDNA in GBM39 neurospheres (Fig. 4l and Methods). Targeted methylation of retention elements substantially reduced the growth and viability of GBM39 cells, as expected following the loss and silencing of ecDNA-encoded oncogenes that are key drivers of cancer cell survival (Extended Data Fig. 10b,c). Owing to the acute loss of viability in cells with ecDNA retention elements targeted by CRISPRoff, we were limited to collecting cells at early time points and did not observe a reduction in total ecDNA copy number at 5 days after transfection (Extended Data Fig. 10d). However, when we used imaging to distinguish ecDNA tethering from the effects of oncogene silencing, we found that CRISPRoff targeting of retention elements significantly increased the frequency of cells with untethered ecDNA foci and reduced nuclear ecDNA compared with nontargeting controls (Fig. 4m and Extended Data Fig. 10e,f). To further ensure that ecDNA depletion is due to silencing of retention element function rather than negative selection due to transcriptional silencing of the oncogene, we leveraged our episome retention assay. In vitro CpG methylation of a plasmid containing a single retention element, but no coding genes, completely ablated the episomal retention conferred by this genetic element (Fig. 4n). We corroborated these data by live-cell imaging, which independently showed that methylation decreased physical colocalization of plasmid DNA with mitotic chromosomes during DNA segregation (Extended Data Fig. 10g). Together, our results show that episomal retention of DNA is promoted by retention elements, the hypomethylation of which at CpG sites not only augments oncogene transcription but also enables the molecular interactions required to confer retention of episomal DNA.

## Discussion

ecDNAs are powerful drivers of oncogene expression in human cancers but risk being lost with every cell division. Ensuring its faithful transmission into daughter cells is an evolutionary imperative to achieve ‘episome immortality’. Through genome-wide functional screening, imaging and chromatin profiling, we discovered a new class of pervasive genomic elements that promote retention of ecDNA copies in dividing cells (Fig. 4o). We showed that these retention elements comprise transcriptionally active regions of the human genome and are co-amplified on oncogenic ecDNAs in human cancers. Retention elements physically interact with mitotically bookmarked regions on chromosomes and promote tethering of ecDNA to chromosomes during mitosis. Furthermore, the extrachromosomal retention of these genomic elements is sensitive to methylation at CpG sites, which indicates that molecular interactions that mediate DNA retention can be altered through epigenetic modifications. As ecDNA molecules that contain retention elements should in theory outcompete those that lack them in a cancer cell population, ecDNA retention probably represents a selection process that shapes the size and sequence of amplified DNA in cancer genomes.

We introduce Retain-seq as a mechanism-agnostic platform to discover functional DNA retention elements in human cells. We showed with live-cell imaging that inclusion of a retention element can promote colocalization of episomal DNA with mitotic chromosomes. This result is consistent with the idea that tethering of acentric DNA to chromosomes promotes its retention in the nuclear space of dividing cells. However, we do not rule out orthogonal mechanisms^52^ by which ecDNA can be retained in cells. We recently reported the phenomenon of ecDNA coordinated inheritance, in which multiple ecDNA species in a cell can be inherited together by the same daughter cell during cell division^6^. Concomitant with intermolecular interactions between ecDNA species that facilitate their co-segregation, ecDNA hitchhiking may also occur indirectly if an ecDNA interacts with another ecDNA that contains retention elements. As the composition of retention elements encoded in the ecDNA amplicon may affect the fidelity of its inheritance, the sequence compositions and sizes of ecDNA species are probably a source of variation among ecDNA species and cancer cells.

Our results suggest that retention elements repurpose long-range DNA contacts via mitotic bookmarking for ecDNA hitchhiking. In interphase cells, interactions between enhancers and promoters allow multiple DNA regulatory elements to contact and activate genes up to 1 Mb away on the linear chromosome, typically *in* *cis* on the same chromosome. Large condensates that include Mediator and RNA polymerase II maintain this linkage to facilitate active transcription^53,54^. During mitosis, transcription is silenced and transcription factors dissociate from condensed mitotic chromosomes. However, certain transcription factors and chromatin-binding proteins are retained, which enables prompt resumption of gene expression and cell fate in the daughter cells. Rather than a binary classification, recent studies indicate that many transcription factors continue to dynamically interact with mitotic chromosomes, and mitotic bookmarking factors have longer occupancy time on mitotic chromosomes^37–43^. Thus, ecDNA may tether to chromosomes during mitosis by recapitulating long-range contacts between bookmarked enhancers and promoters, but *in* *trans* across distinct DNA molecules. The repurposing of mitotic bookmarks explains why retention elements are pervasive throughout the human genome and suggests that many, if not most, chromosomal segments that are sufficiently large are capable of becoming persistent ecDNAs provided that they confer selective advantages to cells. Notably, unlike chromosomes, ecDNAs have highly accessible chromatin^55^ and continue to transcribe RNA at the onset of mitosis^6^, which may promote retention^46^. In EBV and papillomavirus, episomes bind BRD4 (refs. ^18,56^) to hitchhike on mitotic chromosomes, whereas in yeast, selfish 2 micron plasmids bind the SWI/SNF complex^57^ for this process. Both BRD4 and SWI/SNF are prominent mitotic bookmarks^58,59^, which implicates a unifying principle for this mechanism. Our discovery that human retention elements require DNA demethylation suggests that ecDNA selection occurs both at the genetic level for oncogene cargo and at the epigenetic level for active retention-element states. We are inclined to think that the more a retention element is active as a promoter and demethylated in its native chromosomal context, the more likely that such element can facilitate retention when liberated as ecDNA. Future systematic functional studies may identify factors that are necessary for ecDNA hitchhiking and confirm the generalizability of retention element behaviour across cell types. Identification of these mediators of ecDNA retention may facilitate the design of new cancer therapies that target the maintenance of oncogene copies.

Together, our work illustrates how a new class of genomic elements promote the retention of ecDNA in actively dividing cancer cells. These genomic elements may drive the selection of amplicon sequences and structures in cancer to affect the process of DNA amplification and evolutionary trajectories of cancer clones. A mechanistic understanding of ecDNA retention may provide insights into how different cancer cell populations use various levels of oncogene copy number changes and how specific ecDNA amplicon sequences are selected in tumours. Beyond oncogene amplification in cancer, our model of extrachromosomal retention of DNA sequences provide a general framework for understanding the minimal unit of DNA maintenance in human cells and may guide the design of synthetic DNA cargos for cellular engineering efforts.

## Methods

### Cell culture

The GBM39 neurosphere cell line has been previously described^60^: it is derived from a patient with glioblastoma undergoing surgery at the Mayo Clinic. The COLO320DM and K562 cell lines were purchased from the American Type Culture Collection (ATCC), and the GM12878 cell line was purchased from the Coriell Institute for Medical Research. The colorectal cancer cell line COLO320DM and the immortalized chronic myelogenous leukaemia cell line K562 were cultured in RPMI 1640 medium with GlutaMAX (Thermo Fisher Scientific, 61870127) supplemented with 10% FBS (Thermo Fisher Scientific, A3840002) and 1% penicillin–streptomycin (Thermo Fisher Scientific, 15140163). GBM39 cells were maintained in DMEM/F12 (Thermo Fisher Scientific, 11320082), B-27 supplement (Thermo Fisher Scientific, 17504044), 1% penicillin–streptomycin, human epidermal growth factor (EGF, 20 ng ml^–1^; Peprotech, AF-100-15), human fibroblast growth factor (FGF, 20 ng ml^–1^; Peprotech, AF-100-18B) and heparin (5 µg ml^–1^; Sigma-Aldrich, H3149). The lymphoblastoid cell line GM12878 was grown in RPMI 1640 with GlutaMAX supplemented with 15% FBS and 1% penicillin–streptomycin. The COLO320DM live-cell imaging line was cultured in DMEM (Corning, 10-013-CV) supplemented with 10% FBS and 1% penicillin–streptomycin–glutamine (Thermo Fisher Scientific, 10378016). GBM39 neurospheres were previously authenticated by the Mischel Laboratory using metaphase DNA-FISH^12^; other cell lines obtained from the ATCC and Coriell were not authenticated. All cell lines tested negative for mycoplasma contamination.

### Analysis of ecDNA hitchhiking in IF–DNA-FISH of anaphase cells

Analysis of ecDNA hitchhiking in IF–DNA-FISH of anaphase cells was performed on raw images used in a previous publication^5^. Mitotic cells were identified using Aurora kinase B, which marks daughter cell pairs undergoing mitosis, as previously described^5,6^. Colocalization analysis for ecDNAs with mitotic chromosomes in GBM39 cells (*EGFR* ecDNA), PC3 cells (ec*MYC*), SNU16 cells (*FGFR2* ecDNA and ec*MYC*) and COLO320DM cells (ec*MYC*) described in Fig. 1 was performed using Fiji (v.2.1.0/1.53c)^61^. Images were split into the FISH colour + DAPI channels, and the signal threshold was manually set to remove background fluorescence. DAPI was used to mark mitotic chromosomes, and FISH signals overlapping with mitotic chromosomes were segmented using watershed segmentation. Colocalization was quantified using the ImageJ-Colocalization Threshold program, and individual and colocalized FISH signals in dividing daughter cells were counted using particle analysis.

### Retain-seq

We cloned random genomic sequences into the pUC19 plasmid backbone for the Retain-seq experiments. pUC19 is a simple, small (about 2.7 kb) vector that lacks a mammalian origin of replication and contains few sequences that could be immunogenic or have mammalian promoter or enhancer activity. Therefore, we considered that pUC19 represents an inert and selectively neutral backbone. Consequently, changes in plasmid persistence can be more confidently ascribed to insert sequences as opposed to backbone components under selection. To generate a pool of random genomic sequences, we first fragmented the gDNA of GM12878 cells via transposition with Tn5 transposase, produced as previously described^62^, in a 50-µl reaction with TD buffer^63^, 50 ng DNA and 1 µl transposase. The reaction was performed at 37 °C for 5 min, and transposed DNA was purified using a MinElute PCR Purification kit (Qiagen, 28006). GM12878 human B lymphoblastoid cells were selected as the genome of origin owing to their relatively low copy-number variability and the presence of an EBV genome as a positive control; the majority of inserts ranged from 600 to 1,300 bp. The resulting mixture of gDNA fragments was then amplified using 500 nM forward (p5_pUC19_SmaI_20bp) and reverse (p7_pUC19_SmaI_20bp) primers using NEBNext High-Fidelity 2× PCR master mix (NEB, M0541L) followed by gel purification of DNA fragments between 400 bp and 1.5 kb. To insert the mixture of gDNA fragments into a plasmid, the pUC19 vector (Invitrogen) was linearized with SmaI, purified using NucleoSpin Gel and PCR Clean-up (Macherey-Nagel, 740609.250) and the genomic fragments were inserted into the backbone using Gibson assembly (New England Biolabs, NEB). The DNA product was electroporated into Endura Competent Cells (Biosearch Technologies, 60242-2) using a MicroPulser electroporator (Bio-Rad; default bacteria setting) following the manufacturer’s protocol, and the resulting mixed episome library was prepared using a HiSpeed Plasmid Maxi Kit (Qiagen, 12663). The analysis of representation of DNA sequences in this mixed episome library and the retained episomes in transfected cells is described below.

COLO320DM and K562 cells were seeded into a 15 cm dish per biological replicate at a density of 1 × 10^7^ cells in 25 ml of medium. GBM39 cells were seeded into a T75 flask at a density of 5 × 10^6^ cells in 25 ml of medium. Each cell line was incubated overnight. COLO320DM, GBM39 and K562 cells were transfected with 15 µg of an input mixed episome library using Lipofectamine 3000 transfection reagent following the manufacturer’s directions. In brief, 1.5 × 10^7^ GM12878 cells were electroporated with 50 µg input mixed episome library using the Neon Transfection system (Thermo Fisher Scientific, MPK5000). The cells were counted, centrifuged at 300*g* for 5 min and washed twice with PBS before resuspension in Neon Resuspension buffer to a density of 4.2 × 10^6^ in 70 µl of buffer. The input mixed episome library was also diluted to a density of 14 µg in 70 µl with Neon Resuspension buffer. Next, 70 µl of cell suspension and 70 µl of library were mixed and electroporated according to the manufacturer’s instructions using a 100 µl Neon pipette tip under the following settings: 1,200 V, 20 ms, 3 pulses. Five electroporation reactions were pooled per replicate of GM12878 Retain-seq screens.

Cells were incubated for 2 days before the first subculture to allow recovery from transfection, and then subcultured every 3–4 days afterwards as dictated by the doubling time of each cell line. Once each cell line reached a count of 100–400 million cells per replicate, we collected all but 10 million cells, which were maintained in culture and passaged in the same manner until all subsequent time points had been collected (for a maximum of 3 time points per cell line). Thus, COLO320DM cells were collected at days 7, 14 and 21 after transfection, with a total cell count of approximately 4 × 10^8^ cells at each time point, per replicate. GBM39 cells were collected at days 10, 20 and 30, with total cell counts of approximately 1.5 × 10^8^ per replicate. K562 cells were collected at days 6, 12 and 18, with cell counts of approximately 4.5 × 10^8^ per replicate. GM12878 cells were collected at day 12, with a cell count of approximately 2 × 10^8^.

The output plasmid library was extracted using a HiSpeed Plasmid Maxi kit (Qiagen, 12663) and concentrated to a final volume of 50 µl by isopropanol precipitation. DNA was precipitated with a 1:10 volume of 3 M sodium acetate and 2 volumes of isopropanol, chilled at 4 °C for 10 min and centrifuged at 15,000*g* for 15 min at 4 °C. The pellet was washed with 500 µl ice-cold 70% ethanol and dissolved in 50 µl Buffer EB (Qiagen, 19086).

To enrich for input mixed episome library inserts, a preliminary PCR amplification (PCR1) of 10 cycles using primers (at 500 nM) annealing to the pUC19 vector (forward: pUC19_SmaI_5prime_fwr; reverse: pUC19_SmaI_3prime_rev) were performed on the concentrated DNA using NEBNext High-Fidelity 2× PCR master mix (NEB, M0541L). Each PCR1 reaction used a maximum of 2 µg concentrated DNA as template, with reactions assembled successively until all concentrated DNA was consumed; all reactions for a given sample were pooled following PCR1 and purified using a NucleoSpin Gel & PCR Clean-up kit (Macherey-Nagel, 740611), resulting in PCR product 1. Owing to variabilities in the insert size and the amount of retained plasmid DNA in the output library, artificial over-representation of fragments caused by PCR overcycling represented a concern for subsequent sequencing. Thus, we used qPCR to identify the cycle before saturation and halted amplification at this point. For qPCR, 50 ng of DNA from PCR product 1, NEBNext High-Fidelity 2× PCR master mix, 500 nM forward and reverse primers (forward: p5_adapter_only; reverse: p7_adapter_only) and 1 µl of 25× SYBR Green I (diluted from 10,000× stock; Thermo Fisher Scientific, S7563) were used in a 50 µl reaction. The SYBR Green signal of amplification products was measured in technical triplicates per reaction using a Lightcycler 480 (Roche) and plotted against the cycle number to identify the PCR cycle before saturation. According to the cycle numbers identified by this qPCR step, we then performed PCR2 by amplifying PCR product 1 (50 ng DNA) using the same primers as for the qPCR with the following number of cycles: 5, 10 and 12 PCR cycles for days 7, 14 and 21, respectively, of the COLO320DM experiment; 5, 11 and 18 PCR cycles for days 10, 20 and 30, respectively, of the GBM39 experiment; 5, 11 and 17 PCR cycles for days 6, 12, and 18, respectively, of the K562 experiment; and 10 PCR cycles for day 12 of the GM12878 experiment. We also collected a day-17 time point from the GM12878 experiment (amplified using 16 PCR cycles) that was specifically used to study retention of the EBV FR element, as this time point was assumed to be more comparable to the second time point in other cell lines. Next, output DNA from this step (PCR product 2) was purified using a MinElute PCR Purification kit (Qiagen, 28006) and then transposed with Tn5 transposase produced as previously described^62^ in a 50 µl reaction with TD buffer^63^, 50 ng DNA (PCR product 2) and 1 µl transposase. The reaction was performed at 50 °C for 5 min, and transposed DNA was purified using a MinElute PCR Purification it (Qiagen, 28006). The above PCR steps and transposition were also carried out on the input mixed episome library originally used for cell transfection, but with 25 ng of input mixed episome library for PCR1. According to the cycle numbers identified by this qPCR step, we then amplified PCR product 1 (1 ng DNA) over 9 PCR cycles (PCR2). Finally, the previous PCR steps and transposition were also performed on a dilution series of 10 ng, 1 ng, 0.1 ng, and 0.01 ng of input mixed episome library as PCR1 template DNA to standardize analysis of screen output across varying DNA amounts.

Sequencing libraries were generated using five rounds of PCR amplification on the transposed PCR product; 2 using NEBNext High-Fidelity 2× PCR master mix (NEB, M0541L) with primers with i5 and i7 indices, purified using a SPRIselect reagent kit (Beckman Coulter, B23317) with left-sided size selection (1.2×), and quantified using Agilent Bioanalyzer 2100. Libraries were diluted to 4 nM and sequenced on an Illumina NovaSeq 6000 platform.

Primer sequences are listed in Supplementary Table 2.

### Retain-seq analysis

Adapter content in sequenced episome library reads were trimmed using Trimmomatic (v.0.39)^64^. Reads were aligned to the hg19 genome using BWA MEM (v.0.7.17-r1188)^65^ and PCR duplicates were removed using MarkDuplicates in Picard (v.2.25.3). Read counts were then obtained for 1-kb windows across the reference hg19 genome using bedtools (v.2.30.0). Windows with fewer than 10 reads in 1 kb in the input episome library were filtered out.

Next, read counts were normalized to total reads and scaled to counts per million. We filtered out blacklist regions of the genome^66^ and windows with extreme outlier read counts in the input episome library (more than three standard deviations above the mean read count). To determine how genome coverage is affected by the input DNA amount, we measured read counts of 1-kb genomic bins from sequencing of serial dilutions of the input episome library. This serial dilution experiment showed consistent representation of DNA sequences down to 0.1 ng of input DNA, at which the genome representation was nearly identical to 1 ng and 10 ng of input DNA in the top 50% of genomic bins (Extended Data Fig. 1b; 0.01 ng showed substantial library dropout and signs of skewing). Therefore, we focused our subsequent analyses of Retain-seq data on time points at which at least 50% of genomic bins are represented (that is, above 10 reads in a 1-kb window). Data from GBM39 cells at day 30 showed low genome representation and were excluded from subsequent analyses. Data from K562 cells at day 18 showed a large drop in genome representation and were excluded from subsequent analyses (Extended Data Fig. 2a).

We then calculated the log_2_[fold change] of each genomic window in each sample over the input episome library by dividing the respective counts per million followed by log-transformation. Regions of the background genome with copy-number amplification in cells that retain the episome library can increase the background sequencing reads that align to those regions. To remove such background genomic noise, we calculated the median log_2_[fold change] values of the neighbouring windows ±5 kb from each 1-kb window and normalized the log_2_[fold change] of each 1-kb window to its corresponding neighbour average. Thus, any enriched episome sequence was required to have increased signal both compared with the input level and with its neighbouring sequences in its position in the reference human genome. *z* scores were calculated using the formula *z* = (*x *– *m*)/s.d., where *x* is the log_2_[fold change] of each 1-kb window, *m* is the mean log_2_[fold change] of the sample, and s.d. is the standard deviation of the log_2_[fold change] of the sample. *z *scores were used to compute upper-tail *P* values using the normal distribution function, which were adjusted with p.adjust in R (v.3.6.1) with the Benjamini–Hochberg procedure to produce false discovery rate values. To identify episomes enriched in various cell lines, we identified 1-kb windows with false discovery rate values of <0.1 in two biological replicates at any of the time points for sample collection.

### Plasmid cloning

To individually validate retention elements, pUC19 (empty vector) was digested with SmaI. Then, the following six retention element sequences were PCR amplified via a two-step nested PCR from gDNA derived from the GM12878 cell line: RE-A, chromosome 7 (55,321,959–55,323,480); RE-B, chromosome 7 (55,432,848–55,434,854); RE-C, chromosome 8 (127,725,819–127,727,938); RE-D, chromosome 7 (56,032,209–56,033,389); RE-E, chromosome 7 (55,086,476–55,088,263); and RE-F, chromosome 7 (55,639,062–55,640,378). Each retention element was inserted into the empty vector by Gibson assembly using NEBuilder HiFi 2× DNA Assembly master mix (NEB, E2621L) in accordance with the manufacturer’s protocol. The resulting plasmids were named pUC19_RE-A, pUC19_RE-B, pUC19_RE-C, pUC19_RE-D, pUC19_RE-E and pUC19_RE-F, respectively.

To clone pUC19 plasmids containing the EBV tether (pUC19_FR) or the entire viral origin (tether and replicator; pUC19_oriP), the viral tether (FR element; EBV: 7,421–8,042) and viral origin (*oriP*; EBV: 7,338-9,312) sequences were PCR-amplified using the pHCAG-L2EOP plasmid (Addgene, 51783)^67^ as a template and inserted into SmaI-digested pUC19 by Gibson assembly.

To clone pUC19 plasmids with two or three copies of a retention element (RE-C, chromosome 8 (12,7725,819–127,727,938); pUC19_2RE and pUC19_3RE), we digested pUC19_RE-C with HindIII and inserted a second copy of the retention element (amplified by PCR primers pUC19_2RE forward and pUC19_2RE reverse) by Gibson assembly to generate pUC19_2RE. To generate pUC19_3RE (three copies of the retention element), pUC19_2RE was digested with SacI and a third copy of the retention element (amplified by PCR primers pUC19_3RE forward and pUC19_3RE reverse) was inserted by Gibson assembly.

To clone the pUC19 plasmid containing the CMV promoter (pUC19_CMV), the CMV promoter was PCR-amplified (primers pUC19_CMV forward and pUC19_CMV reverse) using the pGL4.18 CMV-Luc plasmid (pGL4; Addgene, 100984)^68^ as a template and inserted into HindIII-digested pUC19 by Gibson assembly. To clone the pGL4 vector containing a retention element (RE-C, chromosome 8 (127,725,819–127,727,938); pGL4_RE-C), we digested pGL4 with MfeI and BamHI for the backbone and PCR-amplified the retention element sequence from GM12878 gDNA (primers pGL4_RE1 forward and pGL4_RE1 reverse). The PCR product was gel purified, digested with BsaI and BamHI, and ligated to the vector backbone using the DNA Ligation Kit v.2.1 (Takara Bio, 6022) following the manufacturer’s protocol.

For cloning individual overlapping tiles of a retention element (RE-C, chromosome 8 (127,725,819–127,727,938), tiles were each 500 bp in length (with the first 250 bp overlapping with the previous tile and the latter 250 bp with the subsequent tile), and each tile was amplified by PCR using pUC19_RE-C as a template. pUC19 was digested with SmaI and each tile sequence was inserted by Gibson assembly.

The plasmids for live-cell imaging were designed on the basis of a previously published pGL4 vector for a dual luciferase assay^23^. The vector contains a retention element (chromosome 8, (128,804,981–128,806,980), hg19) overlapping with the *PVT1* promoter termed RE-G. To insert LacO repeats for imaging, we first inserted multiple enzyme sites (GTCGACTGTGCTCGAGAACACGGATCCTATGCTCGTACG) by Gibson assembly following digestion with BamHI. Next, the vector was digested with SalI and Bsiwi and ligated with an array of 256 LacO copies that was obtained through the digestion of a pLacO-ISce1 plasmid (Addgene, 58505)^69^ with SalI and Acc65I. To create a control plasmid that does not contain the retention element, the vector was digested with KpnI and BglII. The plasmid sequences were verified by Sanger sequencing. The LacO repeats in the plasmids were further verified by agarose gel because of its large size. All enzymes and Gibson assembly mix were purchased from NEB. All primer sequences are listed in Supplementary Table 2.

### qPCR analysis of plasmid retention

To assess the retention of individual plasmids transfected into cells, we seeded K562 or COLO320DM cells into 6-well plates at a density of 3 × 10^5^ cells in 3 ml of medium per well and incubated the cells overnight. The next morning, cells were transfected with 0.5 µg plasmid per well using Lipofectamine 3000 transfection reagent (Thermo Fisher Scientific) following the manufacturer’s protocol. In total, 6 × 10^5^ GM12878 cells were electroporated with 2 µg plasmid per well using a Neon transfection system. Cells were counted, centrifuged at 300*g* for 5 min and washed twice with PBS before resuspension in Neon resuspension buffer to a density of 4.2 × 10^5^ in 7 µl of buffer. The plasmid was also diluted to a density of 1.4 µg in 7 µl with Neon resuspension buffer. Next, 7 µl of cell suspension and 7 µl of plasmid were mixed and electroporated according to the manufacturer’s instructions using a 10 µl Neon pipette tip under the following settings: 1,200 V, 20 ms, 3 pulses. Two electroporation reactions were pooled per replicate and plated into a 12-well plate in 1.5 ml medium per well. Cell cultures were split every 2–4 days and fresh medium was added. To quantify plasmid DNA in cells at various time points, gDNA was extracted from cells using a DNeasy Blood & Tissue kit (Qiagen, 69504). qPCR was performed in technical duplicates using 50–100 ng gDNA, 2× LightCycler 480 SYBR Green I master mix (Roche, 04887352001) and 125 nM forward and reverse primers (primers pUC19_F and pUC19_R, annealing to the pUC19 vector backbone; for plasmids with the pGL4 vector backbone, primers pGL4_F and pGL4_R were used). Relative plasmid DNA levels were calculated by normalizing to *GAPDH* controls (primers GAPDH_F and GAPDH_R). DNA levels were further normalized to the day 2 levels to account for variability in transfection efficiencies and to cells transfected with an empty plasmid vector control. *P* values were calculated in R using Student’s *t*-tests by comparing the relative fold change of biological replicates at various time points with respect to the input levels at day 2. Primer sequences are listed in Supplementary Table 2.

### Analysis of potential genomic integration of plasmids

COLO320DM cells were seeded into 2 wells of a 6-well plate, transfected with 0.5 µg of pUC19 or pUC19_RE-C per well and passaged as described in the section ‘qPCR analysis of plasmid retention’. At day 8, high-molecular-mass gDNA was extracted from cells with a Puregene Cell Core kit (Qiagen, 158046) and long-read sequencing libraries were prepared using a Ligation Sequencing Kit v.14 (Oxford Nanopore Technologies, SQK-LSK114) in accordance with the manufacturer’s protocol. Libraries were loaded onto R10.4.1 flow cells (Oxford Nanopore Technologies, FLO-PRO114M) and sequenced on a PromethION platform (Oxford Nanopore Technologies). Basecalling from raw POD5 data was performed using the high accuracy DNA model in Dorado (Oxford Nanopore Technologies, v.0.5.2). Fastq files were generated using samtools bam2fq (v.1.6)^70^, aligned to a custom reference (hg19_pUC19) comprising the pUC19 sequence appended to the hg19 genome using minimap2 (v.2.17)^71^ and sorted and indexed using samtools. Alignments shorter than 1 kb and with mapping quality below 60 were discarded. Structural variants were then called using Sniffles (v.2.2)^72^ with the hg19_pUC19 reference and the following parameters: “--allow-overwrite --output-rnames --non-germline --long-ins-length 3000”. Integration events were identified from Sniffles output (.vcf) as Breakends (Translocations) between the pUC19 sequence and chromosomes.

### ENCODE data integration

To perform meta-analysis of protein-binding sites in retention elements, ENCODE data were downloaded in bigWig format using the files.txt file returned from the ENCODE portal (https://www.encodeproject.org) and the following command: “xargs -n 1 curl -O -L <files.txt”. Retention element coordinates in K562 cells were converted from the h19 build to the hg38 build using the UCSC LiftOver tool (R package liftOver, v.1.18.0). To plot heatmaps of protein binding in retention elements, we used the ‘computeMatrix’ function in deepTools (v.3.5.1) with the ‘scale-regions’ mode, specified each ‘bigWig’ file using “--scoreFileName”, and a.bed file containing hg38 retention element coordinates using “--regionsFileName”, along with the following parameters: “--regionBodyLength 5000 --beforeRegionStartLength 5000 --afterRegionStartLength 5000 --binSize 20 –skipZeros”. Each resulting matrix was aggregated by computing column means using the colMeans function in R and rescaled to 0–1 using the ‘rescale’ function in the scales (v.1.3.0) package in R.

To analyse overlap of various genomic annotation classes in retention elements, coordinates of each genomic annotation type were first obtained using the R packages TxDb.Hsapiens.UCSC.hg19.knownGene (genes; v.3.2.2) and TxDb.Hsapiens.UCSC.hg19.lincRNAsTranscripts (lncRNAs; v.3.22). ‘All promoters’ comprised sequences 1,500 bp upstream to 200 bp downstream from the TSS for all transcripts in the TxDb objects, extracted using the ‘promoters’ function. 5′ UTR, 3′ UTR, intron and exon sequences were extracted using the ‘fiveUTRsByTranscript’, ‘threeUTRsByTranscript’, ‘intronicParts’ and ‘exonicParts’, functions, respectively, whereas coding and lncRNA promoters were each subsets of the total promoters list. Downstream intergenic regions represent nongenic sequences within 1,500 bp of each TTS, whereas distal intergenic regions were classified as nongenic sequences beyond 1,500 bp of the TSS and 1,500 bp of the TTS. Coordinates were computed using the ‘flank’ and ‘setdiff’ functions in the R package GenomicRanges (v.1.46.1).

To analyse enrichment of transcription-factor-binding sites in retention elements, uniformly processed transcription factor ChIP–seq data (aligned to the hg38 genome) from the K562 cell line were downloaded as a batch from the Cistrome Data Browser (Cistrome DB)^73^. Datasets that failed to meet more than one of the following quality thresholds were excluded: raw sequence median quality score (FastQC score) ≥25; ratio of uniquely mapped reads ≥0.6; PBC score ≥80%; union DNase I hypersensitive site overlap of the 5,000 most significant peaks ≥70%; number of peaks with fold change above 10 ≥500; and fraction of reads in peaks ≥1%. Individual ChIP–seq datasets were imported as GenomicRanges (v.1.46.1) objects from narrowPeak or broadPeak files. For transcription factors with multiple ChIP–seq datasets, datasets were aggregated into a union peak set for subsequent analyses. To identify transcription factors that were enriched for binding in retention elements relative to random genomic intervals, a fold change value was computed for each transcription factor comparing the percentage of retention element intervals overlapping with at least one transcription factor ChIP–seq peak (>50% peak coverage) against the percentage of overlapping 1-kb genomic bins. *P* values were computed in R (function ‘phyper’) using hypergeometric tests for over-representation and adjusted for multiple comparisons with the Bonferroni correction.

### Origins of replication overlap

Coordinates (in the hg19 reference) of origins of replication identified in the K562 cell line across five replicates of SNS-seq were published in another study^74^ and deposited into the NCBI Gene Expression Omnibus (GEO) under accession GSE46189. Retention elements or 1-kb genomic bins were considered overlapping if an origin of replication covered at least 25% of the queried interval (calculated in R using the package GenomicRanges, v.1.46.1). The enrichment *P *value was computed in R using a hypergeometric test for over-representation.

### GRO-seq analysis

GRO-seq data of COLO320DM were published in another study^75^ and deposited into the NCBI GEO under accessions GSM7956899 (replicate 1) and GSM7956900 (replicate 2). The subset of retention element coordinates from the COLO320DM, GBM39 or K562 cell lines located in the amplified intervals of the COLO320DM ecDNA was divided into three categories on the basis of overlap with genomic annotations: (1) retention elements located entirely in coding gene promoters (within 2 kb of a coding gene TSS); (2) retention elements located elsewhere within the limits of coding genes; and (3) retention elements located in noncoding regions. Coordinates of these retention elements were then converted from the hg19 build to hg38 build using the UCSC liftOver package (v.1.18.0) in R. GRO-seq signals within 3 kb of the midpoint of each retention element were presented in separate heatmaps using the EnrichedHeatmap package (v.1.24.0) for each strand and for each retention element category.

### Motif enrichment

A curated collection of human motifs from the CIS-BP database^76^ (‘human_pwms_v2’ in the R package chromVARmotifs, v.0.2.0)^77^ was first matched to the set of 1-kb bins spanning the hg19 reference to identify all such intervals of the human genome containing instances of each motif. Enrichment of each motif in retention elements was then calculated as a log_2_[fold change] of the fraction of retention element intervals (identified by Retain-seq in each cell type) containing motif instances compared with all genomic intervals.

### Live-cell imaging

The live-cell imaging cell line was engineered from COLO320DM cells obtained from the ATCC, as described in a previous publication^6^. TetO ecDNAs were labelled with TetR-mNeonGreen. On the basis of the overlap between MYC and TetO FISH foci in metaphase spreads, 50–80% of ecDNA molecules in a given cell were typically labelled (Extended Data Fig. 6a). The cells were further infected with the LacR-mScarlet-NLS construct and sorted for mScarlet-positive cells to enable stable expression of LacR-mScarlet protein. These cells were then subjected to nucleofection with one of the following plasmids: a control plasmid with LacO repeats; a plasmid containing a retention element (RE-G) with LacO repeats; or an in vitro CpG-methylated retention element (RE-G) plasmid with LacO repeats. Specifically, 1 μg of plasmid was nucleofected into 400,000 cells following the standard nucleofection protocol from Lonza (Nucleofection code, CM-138) to visualize plasmid signals. Cells were seeded onto 96-well glass-bottom plates (Azenta Life Sciences, MGB096-1-2-LG-L) (coated with 10 μg ml^–1^ poly-d-lysine; Sigma-Adrich, A-003-E) immediately after nucleofection and were imaged 2 days later. FluoroBrite DMEM (Gibco, A1896701) supplemented with 10% FBS and 1× GlutaMAX, along with 1:200 Prolong Live antifade reagent (Invitrogen, P36975), was replenished 30 min before time-lapse imaging. Cells were imaged on a top-stage incubator (Okolab) fitted onto a Leica DMi8 wide-field microscope with a ×63 oil objective, and the temperature (37 °C), humidity and CO_2_ (5%) were controlled throughout the imaging experiment. *z* stack images were acquired every 30 min for a total of 4–18 h. The images were processed using Small Volume Computational Clearing before maximum-intensity projections were made for all frames.

### Live-cell imaging analysis

Maximum-intensity projections were exported as TIFF files from the .lif files using ImageJ. To analyse colocalization of LacR–LacO–plasmid foci or TetR–TetO–*MYC* ecDNA foci with mitotic chromosomes during anaphase, images of cells entering anaphase and telophase were exported for mitotic cells that had showed at least five distinct plasmid foci at the beginning of mitosis. The exported images were split into the different colour channels, and the signal threshold was manually set to remove background fluorescence using Fiji (v.2.1.0/1.53c)^61^. Fluorescence signals were segmented using watershed segmentation. The H2B-emiRFP670 signal was used to mark the boundaries of mitotic chromosomes of dividing daughter cells. All colour channels except H2B were stacked, and regions of interest (ROIs) were manually drawn to identify the two daughter cells, and a third ROI was drawn around the space occupied by the pair of dividing daughter cells. Next, the colour channels were split again and image pixel areas occupied by fluorescence signals were analysed using particle analysis. Fractions of ecDNAs colocalizing with mitotic chromosomes were estimated by fractions of FISH pixels in the ROIs of daughter cell chromosome.

To perform time-resolved DNA segregation analysis, TIFF files were analysed using Aivia (v.12.0.0) by first segmenting the condensed chromatin (labelled by H2B- emiRFP670), TetR–TetO–*MYC* foci and LacR–LacO–plasmid foci of the mitotic cell, using a trained pixel classifier that recognizes each of the elements. Each segmented chromatin and focus of interest was then manually selected and output as an object. The relative distance of each focus to its corresponding periphery of the segmented chromatin was output using the Object Relation Tool by setting the ‘TetR/PVT1’ object as the primary set and its corresponding ‘Chromatin’ object as the secondary set using default settings. The resulting data were exported to R (v.3.6.1). TetR–TetO–*MYC* foci or LacR–LacO–plasmid foci with more than 75% overlapping area with the ‘Chromatin’ object were considered colocalized, and their relative distances to their corresponding segmented chromatin were replaced with 0. For each dividing cell, the fractions of plasmid or ecDNA foci colocalizing with mitotic chromosomes were calculated.

### Hi-C

For mitotic Hi-C of COLO320DM cells, cells were seeded into a 6 cm dish at a density of 0.5 × 10^6^ cells in 8 ml RPMI medium (11875-119) containing 10% fetal bovine serum (Fisher Scientific, SH30396.03) and 1% penicillin–streptomycin (Gibco, 15140-122) and the cells were incubated overnight. Nocodazole (M1404-10MG) was dissolved in DMSO and added directly to the cells in the medium to reach a final concentration of 100 ng μl^–1^ (8 μl of 100 ng ml^–1^ nocodazole was added to 8 ml RPMI medium). After 16 h of nocodazole treatment, both suspension and adherent cells were collected for Hi-C analysis and flow cytometry analysis for cell cycle staining using propidium iodide (Invitrogen, 00699050). Flow cytometry verified that the cell population consisted mainly of cells with 4*n* DNA content after mitotic arrest. For interphase Hi-C of GBM39 (GBM39ec) cells, GBM39 cells were cultured as described above (section ‘Cell culture’).

To perform each Hi-C experiment, 10 million cells were fixed in 1% formaldehyde in aliquots of 1 million cells each for 10 min at room temperature and combined after fixation. We performed the Hi-C assay following a standard protocol to investigate chromatin interactions^78^. Hi-C libraries were sequenced on an Illumina HiSeq 4000 with paired-end 75 bp reads for mitotic Hi-C of COLO320DM cells and an Illumina NovaSeq 6000 with paired-end 150 bp reads for interphase Hi-C of GBM39 cells^79^.

### Hi-C analysis

Paired-end Hi-C reads were aligned to hg19 genome with the Hi-C- Pro pipeline^80^. The pipeline was set to default and set to assign reads to DpnII restriction fragments and filter for valid pairs. The data were then binned to generate raw contact maps, which then underwent ICE normalization to remove biases. Visualization was done using Juicebox (https://aidenlab.org/juicebox/). Hi-C data from asynchronous COLO320DM and GBM39 cells were generated and processed in the same way in parallel with the mitotically arrested cells. Asynchronous COLO320DM cell data were separately published^81^ and deposited into the NCBI GEO under accessions GSM8523315 (replicate 1) and GSM8523316 (replicate 2).

To analyse chromatin interactions with retention elements on ec*MYC*, the combined set of retention elements identified was overlapped with the known ec*MYC* coordinates: chromosome 8, 127,437,980–129,010,086 (hg19). To analyse chromatin interactions with chromosome bookmarked regions, we used previously identified bookmarked regions that retained accessible chromatin throughout mitosis in single-cell ATAC–seq data of L02 human liver cells^37^ and filtered out regions that overlap with the known ec*MYC* coordinates and other ec*MYC* co-amplified regions: chromosome 6, 247,500–382,470; chromosome 8, 130,278,158–130,286,750; chromosome 13, 28,381,813–28,554,499; chromosome 16, 32,240,836–32,471,322; and chromosome 16, 33,220,985–33,538,549. The resulting ec*MYC* retention elements and chromosome bookmarked regions were used as anchors to measure pairwise interactions using APA with the .hic files in Juicer (v.1.22.01) and the ‘apa’ function with 5-kb resolution and the following parameters: “-e -u”. Summed percentile matrices of pairwise interactions from ‘rankAPA.txt’ are reported. Analyses for the *EGFR* ecDNA in the GBM39 cell line were performed in the same manner, using the ecDNA coordinates chromosome 7, 54,830,901–56,117,000 (hg19).

To analyse interactions between ENCODE-annotated classes of regulatory sequences, the retention elements that overlapped with ‘dELS’, ‘PLS’ or ‘pELS’ annotations were categorized as distal enhancers, promoters or proximal enhancers, respectively. Those overlapping with both pELS and PLS annotations were categorized as promoters, whereas those overlapping with both pELS or dELS annotations were categorized as proximal enhancers. To extract Hi-C read counts corresponding to interactions between different classes of elements on ecDNA and chromosomes, the Juicer Tools (v.1.22.01)^82^ dump command was used to extract read count data from the .hic files with 1-kb and 5-kb resolution with ‘observed NONE’. The resulting outputs were converted into GInteractions objects using the InteractionSet (v.1.14.0) package in R. To remove chromosomal regions with increased signal due to copy-number changes (and not occurring on ecDNA), we filtered out chromosomal regions that overlapped with copy-number-gain regions identified in WGS of COLO320DM using the ReadDepth (v.0.9.8.5) package. GInteractions objects containing Hi-C read counts between genomic coordinates in 1-kb resolution were overlapped with a GInteractions object containing pairwise interactions between chromosome bookmarked regions and ec*MYC* retention elements using the findOverlaps function in the InteractionSet package in R. Resulting read counts of these pairwise interactions were used to calculate read counts per kb using the formula: read counts per kb = 1,000 × read counts/size of retention element bin in bp. Read counts per kb of each combination of interactions between different classes of elements were summed and divided by the total number of pairwise interactions belonging to each combination of interactions to obtain read counts per kb per interaction.

### Curation of candidate bookmarking factors

Candidate bookmarking factors were curated from three recently published studies^37,39,83^. Candidate bookmarking factors identified in ref. ^39^ were identified in mouse cells. Their orthologues were identified using the Mouse Genome Informatics database (http://www.informatics.jax.org/downloads/reports/HOM_MouseHumanSequence.rpt), and those not annotated as ‘Depleted’ on mitotic chromosomes were included. Candidate bookmarking factors identified in ref. ^37^ were identified on the basis of single-cell ATAC–seq analysis of mitotic chromosomes. Finally, candidate bookmarking factors identified in ref. ^83^ were selected by focusing on protein factors that met the following criterion: log_2_[(*C* + 1)/(*P* + 1)] > 0, where *C* denotes the mean protein enrichment values in mitotic cells from fractionated chromatin (chromatome), and *P* denotes the mean protein enrichment values in the proteomes of mitotic cells.

### Importance analysis of bookmarking factors

To interrogate whether retention elements contain disproportionately more binding sites of some bookmarking factors than others, we computed importance scores in R for each bookmarking factor to explain the observed set of retention elements. First, we generated 1,000 random permutations of the top 20 most enriched bookmarking factors in retention elements compared with random intervals. For each permuted list, we computed the incremental number of retention elements explained by (containing binding sites of) each bookmarking factor in the cumulative distribution. The mean of this value across all permutations represents the importance score for each bookmarking factor.

### CRISPR–Cas9 knockouts of bookmarking factors

Cas9–gRNA ribonucleoprotein (RNP) complexes were first assembled for each gRNA by mixing 30 µM gRNAs (Synthego) targeting *CHD1*, *SMARCE1* and *HEY1* and 2 nontargeting control gRNAs (2 separate guides per target; guide sequences are provided in Supplementary Table 1) separately with 20 µM SpCas9 2NLS Nuclease (Synthego) at a 6:1 molar ratio. Complexes were then incubated for 10 min at room temperature. In brief, COLO320DM cells were counted, centrifuged at 300*g* for 5 min and washed twice with PBS before resuspension in Neon resuspension buffer to a density of 4.2 × 10^5^ in 7 µl of buffer. Next, 7 µl of cell suspension and 7 µl of RNP were mixed and electroporated per reaction according to the manufacturer’s instructions using a 10 µl Neon pipette tip under the following settings: 1,700 V, 20 ms, 1 pulse. Three electroporation reactions were plated for each replicate (2 per condition) into 6-well plates in 3 ml of medium per well.

### IF–DNA-FISH of knockout mitotic cells

About 1 million cells were seeded onto 22 × 22 cm poly-d-lysine-coated coverslips 2 days after transfection. The next day, cells were washed once with 1× PBS and fixed with 4% paraformaldehyde for 10 min at room temperature, followed by permeabilization with 1× PBS–0.25% Triton-X for 10 min at room temperature. Samples were blocked in 3% BSA diluted in 1× PBS for 1 h at room temperature, followed by an overnight incubation at 4 °C with the following primary antibodies: Aurora kinase B antibody (Novus Biologicals, NBP2-50039; 1:1,000); CHD1 (Novus Biologicals, NBP2-14478; 1 μg ml^–1^); HEY1 (Novus Biologicals, NBP2-16818; 1:1,000); and SMARCE1 (Sigma-Aldrich, HPA003916; 1 μg ml^–1^). Cells were washed in 1× PBS and incubated with fluorescently conjugated secondary antibodies (F(ab′)2-goat anti-rabbit IgG (H+L) cross-adsorbed secondary antibody, Alexa Fluor 488 (Invitrogen, A-11070), donkey anti-mouse IgG (H+L) highly cross-adsorbed secondary antibody and Alexa Fluor 647 (Invitrogen, A-31571) at 1:500 for 1 h at room temperature. The samples were then washed in 1× PBS and fixed with 4% paraformaldehyde at room temperature for 20 min. A subsequent permeabilization step using 1× PBS containing 0.7% Triton-X and 0.1 M HCl was performed on ice for 10 min, followed by acid denaturation for 30 min at room temperature using 1.9 M HCl. The samples were then washed once with 1× PBS and then 2× SSC, followed by washes with an ascending ethanol concentration of 70%, 85% and 100% for 2 min each. *MYC* FISH probes (Empire Genomics) were diluted with hybridization buffer and subjected to heat denaturation at 75 °C for 3 mins before applying onto the fully air-dried coverslips for overnight hybridization at 37 °C. The next day, the coverslips were washed once with 0.4× SSC, then with 2× SSC-0.1% Tween 20 and counterstained with DAPI at 50 ng ml^–1^ for 2 min at room temperature. After rinsing in ddH_2_O, the samples were air-dried and mounted onto frosted glass slides with ProLong Diamond antifade mountant (Invitrogen). Samples were imaged on a Leica DMi8 wide-field microscope. *z* stack images were collected and subjected to small volume computational clearing on LAS X.

### Analysis of IF–DNA-FISH of knockout mitotic cells

We first created a CellProfiler (v.4.2.7)^84^ analysis pipeline to quantify protein expression levels after targeted knockdown. In brief, we split each image into four colour channels (DAPI, Aurora kinase B, target protein and ecDNA FISH), and used DAPI to segment nuclei (40–150 pixel units) with global Otsu’s thresholding (two-class thresholding). We then identified cells by starting from the nuclei as seed regions and growing outward using the protein staining signals via propagation with global minimum cross-entropy thresholding. The mean intensity of protein staining in cells was used to determine knockout efficiency of target proteins compared with controls.

Next, we created a CellProfiler analysis pipeline to quantify ecDNA tethering to mitotic chromosomes after protein knockout. In brief, we identified mitotic daughter cell pairs using pairs of cells with Aurora kinase B marking the mitotic midbody as previously described^6^. We segmented nuclei using DAPI as described above and then identified cells by starting from the nuclei as seed regions and growing outward using the protein staining signals via propagation with three-class global Otsu’s thresholding (with pixels in the middle intensity class assigned to the foreground). We separately identified ecDNA foci as primary objects using adaptive Otsu’s thresholding (two-class) and intensity-based declumping. Masks were then created for ecDNA foci overlapping with nuclei (with at least 30% overlap) and ecDNA foci overlapping with cytoplasm (with at least 70% overlap) and defined them as tethered and untethering ecDNA, respectively. The sum of pixel areas was calculated for each group of ecDNA foci and used to calculate tethered ecDNA fractions.

### Evolutionary modelling of ecDNAs

To simulate the effect of retention and selection on ecDNA copy number in growing cell populations, we implemented a new forward-time simulation in Cassiopeia^85^ (https://github.com/YosefLab/Cassiopeia). The simulation framework builds on a previously described forward-time evolutionary model^6^. Specifically, each simulation tracked a the copy-number trajectory of a single ecDNA and was initially parameterized using the following factors: (1) initial ecDNA copy number (denoted as *k*_init_); (2) selection coefficients for cells with no ecDNA (*s*_0_) or at least one copy of ecDNA (*s*_1_); (3) a base birth rate (*λ*_base_ = 0.5); (4) a death rate (*µ* = 0.33); and (5) a retention rate ($\nu \in [0,1]$) that controls the efficiency of passing ecDNA on from generation to generation.

Starting with the parent cell, a birth rate is defined on the basis of the selection coefficient acting on the cell (*s* = *s*_*I*_ or *s*_*I*_, depending on its ecDNA content) as *λ*_1_ =  *λ*_base_  × (1 +* s*). Then, a waiting time to a cell division event is drawn from an exponential distribution: *t*_b_ ∼ exp (–*λ*_1_). Simultaneously, a time to a death event is also drawn from an exponential distribution: *t*_d_ ∼ exp (–*µ*). If *t*_b _< *t*_d_, a cell division event is simulated and a new edge is added to the growing phylogeny with edge length *t*_b_; otherwise, the cell dies and the lineage is stopped. We repeated this process until 25 time units were simulated and at least 1,000 cells were present in the final population.

During cell division, ecDNAs are split among daughter cells according to the retention rate, *v*, and the ecDNA copy numbers of the parent cell. Following previous observations of ecDNA inheritance^5^, ecDNA is divided into daughter cells according to a random binomial process after considering the number of copies of ecDNA that are retained during mitosis. Specifically, with *n*_*i*_ being the number of ecDNA copies in daughter cell $i$ and *N* being the number of copies in the parental cell:

$${n}_{1}=\mathrm{Binomial}(2{N}_{{v}},\,0.5)$$

$${n}_{2}=2{N}_{{V}}-{n}_{1}$$

where Binomial is the binomial probability distribution.

In our experiments, we simulated populations over 25 simulated time units of at least 1,000 cells across ecDNA selection coefficients $${s}_{1}\in [0,0.8]$$ (where *s*_1_ = *0* indicates no selective advantage for cells with ecDNA) and ecDNA retention rates $$\nu \in \{0.5,\,0.6,\,0.7,\,0.8,\,0.9,\,$$$$0.95,\,0.97,\,0.98,\,0.99,\,1.0\}.$$ Selection on cells with no ecDNA was kept at *s*_0_ = 0. We simulated ten replicates per parameter combination and assessed the mean copy number and frequency of ecDNA-positive cells for each time step.

### Analysis of ecDNA sequences in patient tumours

Focal amplification calls predicted by AmpliconArchitect^86^ from tumour samples in The Cancer Genome Atlas and the Pan-cancer Analysis of Whole Genomes cohorts were downloaded from the AmpliconRepository (https://ampliconrepository.org)^87^. A dataset was constructed for ecDNA, BFB and linear amplicons containing the following information for every amplified genomic interval in each amplicon: the corresponding sample, the amplicon number (in that sample), the amplicon ID (assigned in AmpliconRepository), the amplicon classification (ecDNA, BFB or linear), the chromosome, the start and end coordinates, the width, the number of overlapping retention elements and the overlapping oncogenes.

Local retention element density was also computed in R for each amplified interval by dividing the number of retention elements found within 2.5 Mb of the midpoint of the interval by the local window width (5 Mb). Local retention element density was calculated for each amplicon as an average of the local densities of the intervals, weighted by the interval width.

To analyse co-amplification of retention element-negative intervals with retention element-positive intervals, all amplified intervals that lacked retention elements were first identified. If the amplicon corresponding to a given interval contained other intervals with retention elements, then the amplicon was considered co-amplified. Each amplicon was only counted once, regardless of the number of co-amplified retention element-negative intervals. The percentage of amplicons with a co-amplification event was computed for each amplicon class, and *P *values were calculated between classes using a one-sided test of equal proportions.

Predicted ecDNA amplicon intervals containing *EGFR* and *CDK4*, the two most frequently amplified oncogenes in AmpliconRepository samples, were analysed for co-amplification of oncogenes with retention elements. For each oncogene-containing ecDNA interval, 100 random oncogene-containing intervals of the same width were simulated by varying the starting point of the amplified region. For each retention element located within 500 kb of the midpoint of the genomic coordinates of the oncogene, the frequency of inclusion of that retention element in observed oncogene-containing ecDNA intervals was compared with the expected frequency based on the random intervals. Enrichment was computed as a fold change of the observed frequency compared with the expected frequency. *P *values comparing the distributions were calculated in R using a two-sided Fisher’s exact test and adjusted for multiple comparisons with the Benjamini–Hochberg method.

### DNA methylation analysis in nanopore sequencing data

Nanopore sequencing data of GBM39 cells were published in another study^88^ and deposited in the NCBI Sequence Read Archive (SRA) under BioProject accession PRJNA1110283. Bases were called from fast5 files using guppy (Oxford Nanopore Technologies, v.5.0.16) in Megalodon (v.2.3.3), and DNA methylation status was determined using Rerio basecalling models with the configuration file ‘res_dna_r941_prom_modbases_5mC_v001.cfg’ and the following parameters: “--outputs basecalls mappings mod_mappings mods per_read_mods --mod-motif m CG 0 --write-mods-text --mod-output-formats bedmethyl wiggle --mod-map-emulate-bisulfite --mod-map-base-conv C T --mod-map-base-conv Z C”. In downstream analyses, methylation status was computed over 1-kb intervals for retention elements and other matched-size intervals in the *EGFR* ecDNA.

### CRISPRoff

CRISPRoff experiments were performed as described previously^51^, but with modification. In brief, we first cloned a plasmid (cargo plasmid) that simultaneously expresses five guides targeting the five unmethylated retention element sequences found on the *EGFR* ecDNA of the GBM39 cell line under U6 promoters in an array format using a previously described CARGO approach^89^ (guide sequences are provided in Supplementary Table 1). We also cloned a second plasmid (NTC plasmid) containing only a single LacZ*-*targeting guide, with expression also driven by a U6 promoter, as a nontargeting control. The cargo plasmid or the NTC plasmid was co-transfected with the CRISPRoff-v.2.1 plasmid (Addgene, 167981) into 1.5 × 10^7^ GBM39 cells using the Neon transfection system in accordance with the manufacturer’s protocols. In brief, cells were dissociated to a single-cell suspension with 0.5× TrypLE, counted, centrifuged at 300*g* for 5 min and washed twice with PBS before resuspension in Neon resuspension buffer to a density of 4.2 × 10^6^ in 70 µl of buffer; 14 µg CRISPRoff-v2.1 and 7 µg cargo or NTC plasmids were also mixed with Neon resuspension buffer to a total volume of 70 µl. Next, 70 µl of cell suspension and 70 µl of plasmids were mixed and electroporated according to the manufacturer’s instructions using a 100 µl Neon pipette tip under the following settings: 1,250 V, 25 ms, 2 pulses. Five electroporation reactions were pooled per replicate of each condition and cultured in T75 flasks. Cells were further cultured for 2 days, and double-positive cells (mCherry from the cargo plasmid and BFP from CRISPRoff-v2.1, or eGFP from the NTC plasmid and BFP from CRISPRoff-v2.1) were sorted using a BD Aria II instrument. The sorted cells were immediately plated on laminin-coated coverslips in a 24-well plate at a density of 1 × 10^5^ in 450 µl medium in preparation for imaging (see the section ‘CRISPRoff imaging’). The remaining sorted cells were cultured for an additional 3 days and collected for gDNA extraction using a DNeasy Blood & Tissue kit (Qiagen, 69504). ecDNA levels were quantified by WGS (see the section ‘WGS’).

### Imaging validation of CRISPRoff

Two days after sorting, a total of 100,000 cells were seeded onto laminin (10 µg ml^–1^)-coated 12 mm circular coverslips for each transfection condition. Cells were allowed to recover for another 24 h. Cells were washed once with PBS and fixed with 4% paraformaldehyde at room temperature for 10 min, followed by permeabilization with 1× PBS containing 0.5% Triton-X for another 10 min at room temperature. To further enhance fixation and permeabilization, three additional washes with Carnoy’s fixative (3:1 methanol and glacial acetic acid) were performed. The samples were then rinsed briefly with 2× SSC buffer and subjected to dehydration with ascending ethanol concentrations of 70%, 85% and 100%. The coverslips were completely air-dried before the application of a FISH probe mixture (Empire Genomics), which comprised 0.25 µl *EGFR* FISH probe and 4 µl hybridization buffer. The samples were denatured at 75 °C for 3 min and then hybridized overnight at 37 °C in a humidified, dark chamber. Following hybridization, the coverslips were transferred to a 24-well plate and washed once with 0.4× SSC, then 2× SSC 0.1% Tween-20 and then 2× SSC, for 2 min each. DAPI (5 ng ml^–1^) was applied to the samples for 2 min to counterstain nuclei. The samples were then washed with 2× SSC and ddH_2_O before air drying and then mounted with ProLong Diamond. The samples were imaged on a Leica DMi8 wide-field microscope using a ×63 oil objective lens. *z* stacks were acquired (total range = 10 µm, step size of 0.27 µm, 38 steps) and subjected to small volume computational clearing on LAS X software. ImageJ was used to generate maximum-intensity projections for image analysis to quantify total *EGFR* FISH copy number per nucleus.

To quantify total *EGFR* FISH copy number per nucleus, deep-learning-based pixel classifiers were trained on the DAPI and *EGFR* FISH channels to create a smart segmentation and confidence mask, respectively, using Aivia Software (Leica Microsystems). The masks were used to create a protocol to segment FISH foci and assign FISH foci to their corresponding nucleus. The following measurements were exported for quantification: area, circularity and cell ID for nuclei; area and cell ID for FISH foci. Dead cells and mis-segmented cells with a measurement in nuclei with areas >200 and <75, and circularities <0.7, were excluded from the analysis. The number of cells with untethered FISH foci (that is, FISH foci that were not in the nucleus boundaries in viable cells) were manually counted from each transfection condition.

### WGS

WGS libraries were prepared by DNA tagmentation as previously described^6^. We first transposed gDNA from sorted CRISPRoff cells with Tn5 transposase produced as previously described^62^ in a 50-µl reaction with TD buffer^63^, 10 ng DNA and 1 µl transposase. The reaction was performed at 50 °C for 5 min, and transposed DNA was purified using a MinElute PCR Purification kit (Qiagen, 28006). Libraries were generated through 7 rounds of PCR amplification using NEBNext High-Fidelity 2× PCR master mix (NEB, M0541L) with primers bearing i5 and i7 indices, purified using a SPRIselect reagent kit (Beckman Coulter, B23317) with double-sided size selection (0.8× right, 1.2× left), quantified using an Agilent Bioanalyzer 2100, diluted to 4 nM and sequenced on an Illumina Nextseq 550. Adapter content was trimmed from reads using Trimmomatic^64^ (v.0.39), aligned to the hg19 genome using BWA MEM (v.0.7.17-r1188)^65^, and PCR duplicates removed using MarkDuplicates in Picard (v.2.25.3).

### Plasmid in vitro methylation

To measure the effects of CpG methylation on retention element activity on a plasmid, we performed in vitro methylation of plasmids using M.SssI (NEB, M0226M) for 4 h at 37 °C. Plasmids were then extracted using phenol–chloroform and precipitated using ethanol. Purified plasmids were transfected into cells and assayed using qPCR or live-cell imaging as described above in the sections ‘qPCR analysis of plasmid retention’ and ‘Live-cell imaging’, respectively.

### Reporting summary

Further information on research design is available in the Nature Portfolio Reporting Summary linked to this article.

## Online content

Any methods, additional references, Nature Portfolio reporting summaries, source data, extended data, supplementary information, acknowledgements, peer review information; details of author contributions and competing interests; and statements of data and code availability are available at 10.1038/s41586-025-09764-8.

## Supplementary information

Supplementary Tables Supplementary Tables 1 and 2, which contain the CRISPR gRNA sequences and PCR primer sequences used in the study.

Reporting Summary

## Data Availability

Sequencing data generated for this study have been deposited into the NCBI SRA under BioProject accession PRJNA1333946. Coordinates (in the hg19 reference) of origins of replication identified in the K562 cell line were previously derived from SNS-seq data and published alongside those datasets at the NCBI GEO (GSE46189). GRO-seq data of COLO320DM cells were previously generated^75^ and published at the GEO (GSM7956899 (replicate 1) and GSM7956900 (replicate 2)). Asynchronous COLO320DM cell Hi-C data were previously reported^81^ and deposited into the GEO (GSM8523315 (replicate 1) and GSM8523316 (replicate 2)). Nanopore sequencing data of GBM39 cells were generated in a previous study^88^ and deposited in the NCBI SRA under BioProject accession PRJNA1110283. Coordinates (in the hg19 reference) of retention elements identified in the COLO320DM, GBM39 and K562 cell lines are publicly available at Figshare (10.6084/m9.figshare.30239047)^90^.

## Code availability

The ecDNA evolutionary modelling framework used in this study is publicly available through Cassiopeia^85^ at GitHub (https://github.com/YosefLab/Cassiopeia).
