# Supplementary material for: Genetic elements promote retention of extrachromosomal DNA in cancer cells
Source: Nature. 2025 Nov 19;649(8095):152–60. doi: 10.1038/s41586-025-09764-8 (PMC12727538; doi:10.1038/s41586-025-09764-8)
Supplement: Supplementary file 1 — Supplementary Tables 1 and 2, which contain the CRISPR gRNA sequences and PCR primer sequences used in the study. [file 41586_2025_9764_MOESM1_ESM.pdf]

nature portfolio

https://doi.org/10.1038/s41586-025-09764-8

Supplementary information

Genetic elements promote retention of extrachromosomal DNA in cancer cells

In the format provided by the authors and unedited

Genetic elements retain extrachromosomal DNA in dividing cancer cells

Venkat Sankar[1,#], King L. Hung[1,4,#], Aditi Gnanasekar[2,3], Ivy Tsz-Lo Wong[2,3], Quanming Shi[1,5], Katerina Kraft[1], Matthew G. Jones[1], Britney Jiayu He[1], Xiaowei Yan[1], Julia A. Belk[1], Kevin J. Liu[6], Sangya Agarwal[1,5], Sean K. Wang[1], Anton G. Henssen[7,8,9], Paul S. Mischel[2,3*], Howard Y. Chang[1,3,5,10*]

[1] Departments of Dermatology and Genetics, Stanford University, Stanford, CA, USA.

[2] Sarafan ChEM-H, Stanford University, Stanford, CA, USA.

[3] Department of Pathology, Stanford University, Stanford, CA, USA.

[4] Current address: Department of Neuroscience, Scripps Research, La Jolla, CA, USA.

[5] Current address: Amgen Research, South San Francisco, CA, USA.

[6] Program in Cancer Biology, Stanford University, Stanford, CA, USA.

[7] Department of Pediatric Hematology and Oncology, Charité-Universitätsmedizin Berlin, Berlin, Germany.

[8] Berlin Institute of Health, Berlin, Germany.

[9] Experimental and Clinical Research Center, Max Delbrück Center for Molecular Medicine and Charité-Universitätsmedizin Berlin, Berlin, Germany.

[10] Howard Hughes Medical Institute, Stanford University School of Medicine, Stanford, CA 94305, USA.

[#] These authors contributed equally

* Correspondence should be addressed to H.Y.C (howchang@stanford.edu) and P.S.M. (pmischel@stanford.edu)

Table of Contents

Supplementary Table 1: CRISPR guide RNA sequences.

Supplementary Table 2: PCR primer sequences.

Supplementary Tables

Supplementary Table 1.

**Guide RNA target sequence**	**Guide RNA information**
AGCGATGCGACCCTCCGGGA	CRISPRoff cargo guide
TCCATCCGCCCGGTCACCGC	CRISPRoff cargo guide
GGCTCATCCGTGGTCGCCGG	CRISPRoff cargo guide
CCGCTCAACAAGTTCCCCGC	CRISPRoff cargo guide
CGCCATCTTGCTCGGCGCCT	CRISPRoff cargo guide
CCACUUGCCGUGAUAUGAAC	CHD1 KO1
UUAAUUCGCCUAAGAGAACG	CHD1 KO2
UCGACAGAGACAAUCUCGCA	SMARCE1 KO1
UUGAUUCUCCUACCGUGACC	SMARCE1 KO2
AAACCAGUCGAACUCGAAGC	HEY1 KO1
AAUGUGUCCGAGGCCCGCGU	HEY1 KO2
GAACGACUAGUUAGGCGUGUA	Non-targeting control 1 (NTC1) (*Gal4* targeting)
GTGCTGCAAGGCGATTAAGT	Non-targeting control 2 (NTC2) (*LacZ* targeting)

Supplementary Table 2.

**Primer sequence**	**Primer information**
GTCGACTCTAGAGGATCCCCTCGTCG GCAGCGTCAGATGTGTATAAGAGACAG	p5_pUC19_SmaI_20bp
TGAATTCGAGCTCGGTACCCGTCTCGT GGGCTCGGAGATGTGTATAAGAGACAG	p7_pUC19_SmaI_20bp
GTCGACTCTAGAGGATCCCC	pUC19_SmaI_5prime_fwr
TGAATTCGAGCTCGGTACCC	pUC19_SmaI_3prime_rev
TCGTCGGCAGCGTCAGATGTGTATAAGAGACAG	p5_adapter_only
GTCTCGTGGGCTCGGAGATGTGTATAAGAGACAG	p7_adapter_only
ACCATGATTACGCCAATCCAGATGCCTCTCTGGCC	pUC19_2RE forward
ACCTGCAGGCATGCACCTAGGCTTGAACCCCTCCA	pUC19_2RE reverse
GGGGTACCGAGCTCGATCCAGATGCCTCTCTGGCC	pUC19_3RE forward
AAACGACGGCCAGTGCCTAGGCTTGAACCCCTCCA	pUC19_3RE reverse
ACCATGATTACGCCAATCCAGATGCCTCTCTGGCC	pUC19_tile1 forward
ACCTGCAGGCATGCAGATGTGGGTGGGGCCAGATA	pUC19_tile1 reverse
ACCATGATTACGCCATTACAGCTCTTAAGGCGGCG	pUC19_tile2 forward

ACCTGCAGGCATGCAACACCAATCGGCACTCTGTATC	pUC19_tile2 reverse
ACCATGATTACGCCACCACATCCTGCTGATTGGTCC	pUC19_tile3 forward
ACCTGCAGGCATGCATCCACTGGGTGAAGCCAGCT	pUC19_tile3 reverse
ACCATGATTACGCCAGATACAGAGTGCCGATTGGTGT	pUC19_tile4 forward
ACCTGCAGGCATGCAGCGCTGTACTCGATTTCTCG	pUC19_tile4 reverse
ACCATGATTACGCCAAGCTGGCTTCACCCAGTGGA	pUC19_tile5 forward
ACCTGCAGGCATGCACCCTCTCTGGGCTGGCCAAG	pUC19_tile5 reverse
ACCATGATTACGCCAGAAATCGAGTACAGCGCCGG	pUC19_tile6 forward
ACCTGCAGGCATGCATGGTGAGAGGCAGAACTGGC	pUC19_tile6 reverse
ACCATGATTACGCCACCTTGGCCAGCCCAGAGAGG	pUC19_tile7 forward
ACCTGCAGGCATGCAGGCTCTGGGACTCAGCATGAGA	pUC19_tile7 reverse
ACCATGATTACGCCAGCCTCTTGTGCCAGTTCTGC	pUC19_tile8 forward
ACCTGCAGGCATGCACCTAGGCTTGAACCCCTCCA	pUC19_tile8 reverse
ACCATGATTACGCCAGGCATTGATTATTGACTAGT	pUC19_CMV forward
ACCTGCAGGCATGCAAGCTCTGCTTATATAGACCT	pUC19_CMV reverse
CAACAAGGTCTCCAATTGACTAGATTAGCTAGATACAGA GTGTCC	pGL4_RE1 forward
GCAAACGGATCCGTTTCTGGAGAAAGGGAGTAAGTAAG	pGL4_RE1 reverse
ATTTCCGTGTCGCCCTTATT	qPCR pUC19_F
ACCGCTGTTGAGATCCAGTT	qPCR pUC19_R
CACCGTCAAGGCTGAGAAC	qPCR GAPDH_F
TATACCCAAGGGAGCCACAC	qPCR GAPDH_R
CCGCCTCCATCCAGTCTAT	qPCR pGL4_F
CGAACGACGAGCGTGATAC	qPCR pGL4_R
