# Supplementary material for: Genetic elements promote retention of extrachromosomal DNA in cancer cells
Source: Nature. 2025 Nov 19;649(8095):152–60. doi: 10.1038/s41586-025-09764-8 (PMC12727538; doi:10.1038/s41586-025-09764-8)
Supplement: Supplementary file 2 — Reporting Summary [file 41586_2025_9764_MOESM2_ESM.pdf]

nature portfolio

Corresponding author(s): Howard Y. Chang, Paul S. Mischel

Last updated by author(s): 9/29/2025

Reporting Summary

Nature Portfolio wishes to improve the reproducibility of the work that we publish. This form provides structure for consistency and transparency in reporting. For further information on Nature Portfolio policies, see our Editorial Policies and the Editorial Policy Checklist.

Statistics

For all statistical analyses, confirm that the following items are present in the figure legend, table legend, main text, or Methods section.

n/a	Confirmed	
☐	☒	The exact sample size (*n*) for each experimental group/condition, given as a discrete number and unit of measurement
☐	☒	A statement on whether measurements were taken from distinct samples or whether the same sample was measured repeatedly
☐	☒	The statistical test(s) used AND whether they are one- or two-sided<br>*Only common tests should be described solely by name; describe more complex techniques in the Methods section.*
☒	☐	A description of all covariates tested
☐	☒	A description of any assumptions or corrections, such as tests of normality and adjustment for multiple comparisons
☐	☒	A full description of the statistical parameters including central tendency (e.g. means) or other basic estimates (e.g. regression coefficient) AND variation (e.g. standard deviation) or associated estimates of uncertainty (e.g. confidence intervals)
☐	☒	For null hypothesis testing, the test statistic (e.g. *F*, *t*, *r*) with confidence intervals, effect sizes, degrees of freedom and *P* value noted<br>*Give P values as exact values whenever suitable.*
☐	☒	For Bayesian analysis, information on the choice of priors and Markov chain Monte Carlo settings
☒	☐	For hierarchical and complex designs, identification of the appropriate level for tests and full reporting of outcomes
☐	☒	Estimates of effect sizes (e.g. Cohen's *d*, Pearson's *r*), indicating how they were calculated

Our web collection on statistics for biologists contains articles on many of the points above.

Software and code

Policy information about availability of computer code

Data collection

WGS, including Retain-seq
Libraries were diluted to 4 nM and sequenced on the Illumina NovaSeq 6000 platform (Retain-seq) and Illumina Nextseq 550 (WGS after CRISPRoff). Reads were trimmed of adapter content with Trimmomatic65 (version 0.39), aligned to the hg19 genome using BWA MEM66 (0.7.17-r1188), and PCR duplicates removed using Picard's MarkDuplicates (version 2.25.3).

Analysis of potential genomic integration of plasmids
Libraries were loaded onto R10.4.1 flow cells (Oxford Nanopore Technologies, FLO-PRO114M) and sequenced on the PromethION platform (Oxford Nanopore Technologies). Basecalling from raw POD5 data was performed using the High accuracy (HAC) DNA model in Dorado (Oxford Nanopore Technologies, version 0.5.2). Fastq files were generated using samtools bam2fq (version 1.6)71, aligned to a custom reference (hg19_pUC19) comprising the pUC19 sequence appended to the hg19 genome using minimap2 (version 2.17)72, and sorted and indexed using samtools; alignments shorter than 1 kb and with mapping quality below 60 were discarded. Structural variants were then called using Sniffles (version 2.2)73 using the hg19_pUC19 reference and the following parameters: "--allow-overwrite --output-rnames --non-germline --long-ins-length 3000". Integration events were identified from Sniffles output (.vcf) as Breakends (Translocations) between the pUC19 sequence and chromosomes.

Hi-C
Hi-C libraries were sequenced on an Illumina HiSeq 4000 with paired-end 75 bp reads for mitotic Hi-C of COLO320DM and an Illumina NovaSeq 6000 with paired-end 150 bp reads for interphase Hi-C of GBM3980. Paired-end Hi-C reads were aligned to hg19 genome with the Hi-C- Pro pipeline81. Pipeline was set to default and set to assign reads to DpnII restriction fragments and filter for valid pairs. The data was then binned to generate raw contact maps which then underwent ICE normalization to remove biases. Visualization was done using Juicebox (https://aidenlab.org/juicebox/). Hi-C data from asynchronous COLO320DM and GBM39 cells were generated and processed in the same way

in parallel with the mitotically arrested cells; asynchronous COLO320DM cell data were separately published with Kraft et al. 2024 (bioRxiv) and deposited in NCBI Gene Expression Omnibus (GEO) under accessions GSM8523315 (replicate 1) and GSM8523316 (replicate 2)82.

Data analysis

Analysis of ecDNA hitchhiking in IF-DNA-FISH of anaphase cells
Analysis of ecDNA hitchhiking in IF-DNA-FISH of anaphase cells was performed on raw images used in a previous publication5. Mitotic cells were identified using Aurora kinase B, which identifies daughter cell pairs undergoing mitosis, as previously described5,6. Colocalization analysis for ecDNAs with mitotic chromosomes in GBM39 cells (EGFR ecDNA), PC3 cells (MYC ecDNA), SNU16 cells (FGFR2 and MYC ecDNAs) and COLO320DM cells (MYC ecDNA) described in Figure 1 was performed using Fiji (v.2.1.0/1.53c)62. Images were split into the FISH color + DAPI channels, and signal threshold set manually to remove background fluorescence. DAPI was used to mark mitotic chromosomes; FISH signals overlapping with mitotic chromosomes were segmented using watershed segmentation. Colocalization was quantified using the ImageJ-Colocalization Threshold program and individual and colocalized FISH signals in dividing daughter cells were counted using particle analysis.

Retain-seq analysis
Sequenced episome library reads were trimmed of adapter content with Trimmomatic65 (version 0.39), aligned to the hg19 genome using BWA MEM66 (0.7.17-r1188), and PCR duplicates removed using Picard's MarkDuplicates (version 2.25.3). Read counts were then obtained for 1-kilobase windows across the reference hg19 genome using bedtools (v.2.30.0). Windows with fewer than 10 reads within 1 kb in the input episome library were filtered out.
Next, read counts were normalized to total reads and scaled to counts per million (CPMs). We filtered out blacklist regions of the genome67 and windows with extreme outlying read counts in the input episome library (more than three standard deviations above the mean read count). To determine how genome coverage is affected by input DNA amount, we measured read counts of 1-kb genomic bins from sequencing of serial dilutions of the input episome library. Based on this serial dilution experiment which showed consistent representation of DNA sequences down to 0.1 ng of input DNA, at which the genome representation was nearly identical to 1 ng and 10 ng of input DNA in the top 50% of genomic bins (Extended Data Figure 1b; 0.01 ng showed substantial library dropout and signs of skewing), we focused our subsequent analysis of Retain-seq on time points at which at least 50% of genomic bins are represented (i.e. above 10 reads within a 1-kb window. GBM39 at day 30 showed low genome representation and was excluded from subsequent analysis. K562 at day 18 showed a large drop in genome representation and was excluded from subsequent analysis; Extended Data Figure 2a).
We then calculated the log2 fold change of each genomic window in each sample over the input episome library by dividing the respective CPMs followed by log-transformation. Regions of the background genome with copy-number amplification in the cells retaining the episome library can elevate the background sequencing reads aligning to those regions. To remove such background genomic noise, we calculated the median log2 fold change values of the neighboring windows +/- 5 kb from each 1-kb window and normalized the log2 fold change of each 1-kb window to its corresponding neighbor average. Thus, any enriched episome sequence was required to have increased signal both compared to the input level as well as its neighboring sequences in its position in the reference human genome. Z scores were calculated using the formula $z = (x-m)/S.D.$, where x is the log2 fold change of each 1-kb window, m is the mean log2 fold change of the sample, and S.D. is the standard deviation of the log2 fold change of the sample. Z scores were used to compute upper-tail P values using the normal distribution function, which were adjusted with p.adjust in R (v.3.6.1) using the Benjamini-Hochberg Procedure to produce false discovery rate (FDR) values. To identify episomes enriched in various cell lines, we identified 1-kb windows with FDR < 0.1 in two biological replicates at any of the time points for sample collection.

ENCODE data integration
To perform meta-analysis of protein binding sites within retention elements, ENCODE data were downloaded in "bigWig" format using the files.txt file returned from the ENCODE portal (https://www.encodeproject.org) and the following command: "xargs -n 1 curl -O -L < files.txt". K562 retention element coordinates were converted from the h19 to hg38 build using the UCSC LiftOver tool (R package liftOver, version 1.18.0). To plot heatmaps of protein binding within retention elements, we used the "computeMatrix" function in deepTools (version 3.5.1) using the "scale-regions" mode, specified each "bigWig" file using "--scoreFileName", and a .bed file containing hg38 retention element coordinates using "--regionsFileName", along with the following parameters: "--regionBodyLength 5000 --beforeRegionStartLength 5000 --afterRegionStartLength 5000 --binSize 20 –skipZeros". Each resulting matrix was aggregated by computing column means using the colMeans function in R and rescaled to 0-1 using the "rescale" function in the scales (version 1.3.0) package in R.
To analyze overlap of various genomic annotation classes within retention elements, coordinates of each genomic annotation type were first obtained using the R packages TxDb.Hsapiens.UCSC.hg19.knownGene (genes; version 3.2.2) and TxDb.Hsapiens.UCSC.hg19.lincRNAsTranscripts (lncRNAs; version 3.22). "All promoters" comprised sequence 1500 bp upstream to 200 bp downstream from the transcription start site for all transcripts in the TxDb objects, extracted using the "promoters" function. 5' UTR, 3' UTR, intron, and exon sequences were extracted using the "fiveUTRsByTranscript", "threeUTRsByTranscript", "intronicParts", and "exonicParts" functions respectively while coding and lncRNA promoters were each subsets of the total promoters list. Downstream intergenic regions represent non-genic sequences within 1500 bp of each transcription termination site while distal intergenic regions were classified as non-genic sequences beyond 1500 bp of the TSS and 1500 bp of the TTS; coordinates were computed using the "flank" and "setdiff" functions in the R package GenomicRanges (version 1.46.1).
To analyze enrichment of transcription factor binding sites within retention elements, uniformly processed transcription factor ChIP-seq data (aligned to the hg38 genome) from the K562 cell line were downloaded as a batch from the Cistrome Data Browser (Cistrome DB)74. Datasets that failed to meet more than one of the following quality thresholds were excluded: raw sequence median quality score (FastQC score) ≥ 25; ratio of uniquely mapped reads ≥ 0.6; PBC score ≥ 80%; union DNase I hypersensitive site overlap of the 5,000 most significant peaks ≥ 70%; number of peaks with fold change above 10 ≥ 500; and fraction of reads in peaks ≥ 1%. Individual ChIP-seq datasets were imported as GenomicRanges (version 1.46.1) objects from narrowPeak or broadPeak files. For transcription factors with multiple ChIP-seq datasets, datasets were aggregated into a union peak set for subsequent analyses. To identify transcription factors that are enriched for binding within retention elements relative to random genomic intervals, a fold change was computed for each transcription factor comparing the percentage of retention element intervals overlapping with at least 1 transcription factor ChIP-seq peak (> 50% peak coverage) against the percentage of overlapping 1 kb genomic bins; p-values were computed in R (function "phyper") using a hypergeometric test for over-representation and adjusted for multiple comparisons by the Bonferroni correction.

Origins of replication overlap
Coordinates (in the hg19 reference) of origins of replication identified in the K562 cell line across 5 replicates of SNS-seq were published with Picard et al. and deposited in NCBI Gene Expression Omnibus (GEO) under accession GSE4618975. Retention elements or 1 kb genomic bins were considered overlapping if an origin of replication covered at least 25% of the queried interval (calculated in R using the package GenomicRanges, version 1.46.1). The enrichment p-value was computed in R using a hypergeometric test for over-representation.

GRO-seq analysis

GRO-seq data of COLO320DM were published with Tang et al. and deposited in NCBI GEO under accessions GSM7956899 (replicate 1) and GSM7956900 (replicate 2)76. The subset of retention element coordinates from the COLO320DM, GBM39, or K562 cell lines located within the amplified intervals of the COLO320DM ecDNA was divided into three categories based on overlap with genomic annotations: 1) retention elements located entirely within coding gene promoters (within 2 kb of a coding gene TSS); 2) retention elements located elsewhere within the limits of coding genes; and 3) retention elements located within noncoding regions. Coordinates of these retention elements were then converted from the hg19 to hg38 build using the UCSC liftOver package (version 1.18.0) in R. GRO-seq signal within 3 kb of the midpoint of each retention element was presented in separate heatmaps using the EnrichedHeatmap package (version 1.24.0) for each strand and for each retention element category.

Motif enrichment
A curated collection of human motifs from the CIS-BP database77 ("human_pwms_v2" in the R package chromVARmotifs, version 0.2.0)78 was first matched to the set of 1 kb bins spanning the hg19 reference to identify all such intervals of the human genome containing instances of each motif. Enrichment of each motif within retention elements was then calculated as a log2(fold change) of the fraction of retention element intervals (identified by Retain-seq in each cell type) containing motif instances compared to all genomic intervals.

Live-cell imaging analysis
Maximum intensity projections were exported as TIFF files from the .lif files using imageJ. To analyze colocalization of LacR-LacO-plasmid foci or TetR-TetO-MYC ecDNA foci with mitotic chromosomes during anaphase, images of cells entering anaphase and telophase were exported for mitotic cells that had showed at least five distinct plasmid foci at the beginning of mitosis. The exported images were split into the different color channels, and signal threshold set manually to remove background fluorescence using Fiji (version 2.1.0/1.53c)62. Fluorescence signals were segmented using watershed segmentation. H2B-emiRFP670 signal was used to mark the boundaries of mitotic chromosomes of dividing daughter cells. All color channels except H2B were stacked and ROIs were drawn manually to identify the two daughter cells, and a third ROI was drawn around the space occupied by the pair of dividing daughter cells. Next, the colour channels were split again and image pixel areas occupied by fluorescence signals were analyzed using particle analysis. Fractions of ecDNAs colocalizing with mitotic chromosomes were estimated by fractions of FISH pixels within the daughter cell chromosome ROIs.
To perform time-resolved DNA segregation analysis, TIFF files were analyzed on Aivia (v.12.0.0) by first segmenting the condensed chromatin (labelled by H2B- emiRFP670), TetR-TetO-MYC foci, and LacR-LacO-plasmid foci of the mitotic cell, using a trained pixel classifier recognizing each of the elements. Each segmented chromatin and focus of interest was then selected manually and output as an object. The relative distance of each focus to its corresponding segmented chromatin's periphery was output using the Object Relation Tool, by setting the 'TetR/ PVT1' object as primary set and its corresponding 'Chromatin' object as secondary set, under default settings. The resulting data were exported to R (v.3.6.1). TetR-TetO-MYC foci or LacR-LacO-plasmid foci with more than 75% overlapping area with the 'Chromatin' object were considered colocalized and their relative distances to their corresponding segmented chromatin were replaced with 0. For each dividing cell, the fractions of plasmid or ecDNA foci colocalizing with mitotic chromosomes were calculated.

Hi-C analysis
To analyze chromatin interactions with retention elements on ecMYC, the combined set of retention elements identified was overlapped with the known ecMYC coordinates: chr8:127437980-129010086 (hg19). To analyze chromatin interactions with chromosome bookmarked regions, we used previously identified bookmarked regions that retained accessible chromatin throughout mitosis in single-cell ATAC-seq data of L02 human liver cells38 and filtered out regions that overlap with the known ecMYC coordinates as well as other ecMYC co-amplified regions: chr6:247500-382470, chr8:130278158-130286750, chr13:28381813-28554499, chr16:32240836-32471322, chr16:33220985-33538549. The resulting ecMYC retention elements and chromosome bookmarked regions were used as anchors to measure pairwise interactions via aggregated peak analysis (APA), using the .hic files in Juicer (v.1.22.01) and the "apa" function with 5-kb resolution and the following parameters: "-e -u". Summed percentile matrices of pairwise interactions from "rankAPA.txt" were reported. Analyses for the EGFR ecDNA in the GBM39 cell line were performed in the same manner, using ecDNA coordinates: chr7:54830901-56117000 (hg19).
To analyze interactions between ENCODE-annotated classes of regulatory sequences, retention elements overlapping with "dELS", "PLS", or "pELS" annotations were categorized as distal enhancers, promoters, or proximal enhancers, respectively; those overlapping with both "pELS" and "PLS" annotations were categorized as promoters; those overlapping with both "pELS" or "dELS" annotations were categorized as proximal enhancers. To extract Hi-C read counts corresponding to interactions between different classes of elements on ecDNA and chromosomes, the Juicer Tools83 (v.1.22.01) dump command was used to extract read count data from the .hic files with 1-kb and 5-kb resolution using "observed NONE". The resulting outputs were converted into GInteractions objects using the InteractionSet (version 1.14.0) package in R. To remove chromosomal regions with elevated signal due to copy-number changes (and not occurring on ecDNA), we filtered out chromosomal regions that overlap with copy-number-gain regions identified in WGS of COLO320DM using the ReadDepth (version 0.9.8.5) package. GInteractions objects containing Hi-C read counts between genomic coordinates in 1-kb resolution were overlapped with a GInteractions object containing pairwise interactions between chromosome bookmarked regions and ecMYC retention elements using the findOverlaps function in the InteractionSet package in R. Resulting read counts of these pairwise interactions were used to calculate read counts per kb using this formula: read counts per kb = 1000 × read counts / size of retention element bin in bp. Read counts per kb of each combination of interactions between different classes of elements were summed and divided by the total number of pairwise interactions belonging to each combination of interactions to obtain read counts per kb per interaction.

Importance analysis of bookmarking factors
To interrogate whether retention elements contain binding sites of some bookmarking factors disproportionately more than others, we computed importance scores in R for each bookmarking factor in explaining the observed set of retention elements. First, we generated 1000 random permutations of the top 20 most enriched bookmarking factors within retention elements compared to random intervals. For each permuted list, we computed the incremental number of retention elements explained by (containing binding sites of) each bookmarking factor in the cumulative distribution. The mean of this value across all permutations represents the importance score for each bookmarking factor.

Analysis of immunofluorescence staining-DNA FISH of KO mitotic cells
We first created a CellProfiler (version 4.2.7)85 analysis pipeline to quantify protein expression levels after targeted knockdown. Briefly, we split each image into four color channels (DAPI, Aurora kinase B, target protein, and ecDNA FISH), and used DAPI to segment nuclei (40-150 pixel units) with global Otsu's thresholding (two-class thresholding). We then identified cells by starting from the nuclei as seed regions and growing outward using the protein staining signals via propagation with global Minimum Cross-Entropy Thresholding. Mean intensity of protein staining in cells was used to determine KO efficiency of target proteins compared with controls.
Next, we created a CellProfiler analysis pipeline to quantify ecDNA tethering to mitotic chromosomes after protein KO. Briefly, we identified mitotic daughter cell pairs using pairs of cells with Aurora kinase B marking the mitotic midbody as previously shown34. We segmented nuclei using DAPI as above and then identified cells by starting from the nuclei as seed regions and growing outward using the protein staining

signals via propagation with three-class global Otsu's thresholding (with pixels in the middle intensity class assigned to the foreground). We separately identified ecDNA foci as primary objects using adaptive Ostu's thresholding (two-class) and intensity-based de-clumping. Masks were then created for ecDNA foci overlapping with nuclei (with at least 30% overlap) and ecDNA foci overlapping with cytoplasm (with at least 70% overlap) and defined as tethered and untethering ecDNA, respectively. The sum of pixel areas was calculated for each group of ecDNA foci and used to calculate tethered ecDNA fractions.

Evolutionary modeling of ecDNAs
To simulate the effect of retention and selection on ecDNA copy-number in growing cell populations, we implemented a new forward-time simulation in Cassiopeia86 (https://github.com/yoseflab/cassiopeia). The simulation framework builds off of the forward-time evolutionary modelling previously described6. Specifically, each simulation tracked a single ecDNA's copy-number trajectory and was initially parameterized by (i) initial ecDNA copy-number (denoted as kinit); (ii) selection coefficients for cells carrying no ecDNA (s0) or at least one copy of ecDNA (s1); (iii) a base birth rate (λ¬_base=0.5); (iv) a death rate (μ=0.33); and (v) a retention rate (v∈[0,1]) that controls the efficiency of passing ecDNA on from generation to generation.
Starting with the parent cell, a birth rate is defined based on the selection coefficient acting on the cell (s = s0 or s1, depending on its ecDNA content) as λ_1=λ_base*(1+s). Then, a waiting time to a cell division event is drawn from an exponential distribution: t_b~exp(- λ_1). Simultaneously, a time to a death event is also drawn from an exponential distribution: t_d~exp(- μ). If t_b<t_d, a cell division event is simulated and a new edge is added to the growing phylogeny with edge length t_b; otherwise, the cell dies and the lineage is stopped. We repeated this process until 25 time units were simulated and at least 1000 cells were present in the final population.
During a cell division, ecDNAs are split amongst daughter cells according to the retention rate, ν, and the ecDNA copy numbers of the parent cell. Following observations of ecDNA inheritance previously reported5, ecDNA is divided into daughter cells according to a random Binomial process, after considering the number of copies of ecDNA that are retained during mitosis. Specifically, with n_i being the number of ecDNA copies in daughter cell i and N being the number of copies in the parental cell:
n_1=Binomial(2Nv,0.5)
n_2=2Nv-n_1
Where Binomial is the binomial probability distribution.
In our experiments, we simulated populations over 25 simulated time units of at least 1000 cells across ecDNA selection coefficients s_1∈[0,0.8] (where s1=0 indicates no selective advantage for ecDNA-carrying cells) and ecDNA retention rates v∈ \{0.5,0.6,0.7,0.8,0.9,0.95,0.97,0.98,0.99,1.0\} . Selection on cells carrying no ecDNA was kept at s0=0. We simulated 10 replicates per parameter combination and assessed the mean copy-number and frequency of ecDNA+ cells for each time step.

Analysis of ecDNA sequences in patient tumors
Focal amplification calls predicted by AmpliconArchitect87 from tumor samples in The Cancer Genome Atlas (TCGA) and Pan-cancer Analysis of Whole Genomes (PCAWG) cohorts were downloaded from AmpliconRepository (https://ampliconrepository.org)88. A dataset was constructed for ecDNA, breakage-fusion-bridge (BFB), and linear amplicons containing the following information for every amplified genomic interval within each amplicon: the corresponding sample, amplicon number (within that sample), amplicon ID (assigned in AmpliconRepository), amplicon classification (ecDNA, BFB, or linear), chromosome, start and end coordinates, width, number of overlapping retention elements, and overlapping oncogenes.
Local retention element density was also computed in R for each amplified interval by dividing the number of retention elements found within 2.5 megabases of the midpoint of the interval by the local window width (5 megabases). Local retention element density was calculated for each amplicon as an average of the intervals' local densities, weighted by interval width.
To analyze co-amplification of retention element-negative intervals with retention element-positive intervals, all amplified intervals lacking retention elements were first identified. If the amplicon corresponding to a given interval contains other intervals with retention elements, then the amplicon was considered co-amplified; each amplicon was only counted once, regardless of the number of co-amplified retention element-negative intervals. The percentage of amplicons bearing a co-amplification event was computed for each amplicon class; p-values were calculated between classes using a one-sided test of equal proportions.
Predicted ecDNA amplicon intervals containing EGFR and CDK4, the two most frequently amplified oncogenes within AmpliconRepository samples, were analyzed for co-amplification of oncogenes with retention elements. For each oncogene-containing ecDNA interval, 100 random oncogene-containing intervals of the same width were simulated by varying the starting point of the amplified region. For each retention element located within 500 kb of the midpoint of the oncogene's genomic coordinates, the frequency of inclusion of that retention element within observed oncogene-containing ecDNA intervals was compared with the expected frequency based on the random intervals. Enrichment was computed as a fold-change of the observed frequency compared to the expected frequency. P-values comparing the distributions were calculated in R using a two-sided Fisher's Exact Test and adjusted for multiple comparisons by the Benjamini-Hochberg method.

DNA methylation analysis in nanopore sequencing data
Nanopore sequencing data of GBM39 was published with Zhu et al.89 and deposited in NCBI Sequence Read Archive (SRA) under BioProject accession PRJNA1110283. Bases were called from fast5 files using guppy (Oxford Nanopore Technologies, version 5.0.16) within Megalodon (version 2.3.3) and DNA methylation status was determined using Rerio basecalling models with the configuration file "res_dna_r941_prom_modbases_5mC_v001.cfg" and the following parameters: "--outputs basecalls mappings mod_mappings mods per_read_mods --mod-motif m CG 0 --write-mods-text --mod-output-formats bedmethyl wiggle --mod-map-emulate-bisulfite --mod-map-base-conv C T --mod-map-base-conv Z C". In downstream analyses, methylation status was computed over 1 kb intervals for retention elements and other matched-size intervals within the EGFR ecDNA.

Imaging validation of CRISPRoff
To quantify total EGFR FISH copy number per nucleus, deep learning-based pixel classifiers were trained on the DAPI and EGFR FISH channels to create a smart segmentation and confidence mask respectively using Aivia Software (Leica Microsystems). The masks were used to create a recipe to segment FISH foci and assign FISH foci to their corresponding nucleus. The following measurements were exported for quantification: Area, Circularity, Cell.ID for nuclei; Area, Cell.ID for FISH foci. Dead cells and mis-segmented cells with a measurement in nuclei with areas greater than 200 and less than 75, and circularities less than 0.7, were excluded from the analysis. Number of cells with untethered FISH foci (i.e. FISH foci that are not within the nuclei boundaries in viable cells) were counted manually from each transfection condition.

For manuscripts utilizing custom algorithms or software that are central to the research but not yet described in published literature, software must be made available to editors and reviewers. We strongly encourage code deposition in a community repository (e.g. GitHub). See the Nature Portfolio guidelines for submitting code & software for further information.

Data

Policy information about availability of data

All manuscripts must include a data availability statement. This statement should provide the following information, where applicable:

- Accession codes, unique identifiers, or web links for publicly available datasets
- A description of any restrictions on data availability
- For clinical datasets or third party data, please ensure that the statement adheres to our policy

Sequencing data generated for this study have been deposited at the NCBI SRA under BioProject accession PRJNA1333946. Coordinates (in the hg19 reference) of origins of replication identified in the K562 cell line were derived from previously generated SNS-seq data and published at the NCBI Gene Expression Omnibus (GEO; GSE46189). GRO-seq data of COLO320DM cells were generated previously and published at the GEO (GSM7956899, replicate 1; GSM7956900, replicate 2). Asynchronous COLO320DM cell Hi-C data were previously deposited at the GEO (GSM8523315, replicate 1; GSM8523316, replicate 2). Nanopore sequencing data of GBM39 was generated in a previous study and deposited in the NCBI Sequence Read Archive (SRA) under BioProject accession PRJNA1110283. Coordinates (in the hg19 reference) of retention elements identified in the COLO320DM, GBM39, and K562 cell lines are publicly available at figshare.

Research involving human participants, their data, or biological material

Policy information about studies with human participants or human data. See also policy information about sex, gender (identity/presentation), and sexual orientation and race, ethnicity and racism.

Reporting on sex and gender	N/A
Reporting on race, ethnicity, or other socially relevant groupings	N/A
Population characteristics	N/A
Recruitment	N/A
Ethics oversight	N/A

Note that full information on the approval of the study protocol must also be provided in the manuscript.

Field-specific reporting

Please select the one below that is the best fit for your research. If you are not sure, read the appropriate sections before making your selection.

☒ Life sciences ☐ Behavioural & social sciences ☐ Ecological, evolutionary & environmental sciences

For a reference copy of the document with all sections, see nature.com/documents/nr-reporting-summary-flat.pdf

Life sciences study design

All studies must disclose on these points even when the disclosure is negative.

Sample size	No statistical methods were used to predetermine the sample size. For sequencing studies, we sequenced DNA from at least 1,000,000 cells which captures much of the genetic heterogeneity in a cell population. All qPCR experiments were performed at least 3 times with biologically independent replicates. Imaging quantifications included 30 or more cells for assessing differences between treatments to capture cell-to-cell variability, and experiments were repeated 2 or more times independently.
Data exclusions	Using a serial dilution experiment for Retain-seq, we determined the degree of genome representation and sequencing read distributions from the episomal DNA libraries and excluded time points which did not meet these criteria (i.e. substantial loss of genome representation).
Replication	Retain-seq experiments in cell lines were replicated independently at least twice at each of various time points to confirm biological effect. Quantitative PCR experiments were performed in at least 3 biological replicates. Transfections to assess genomic integration were performed once per condition, but sufficient cells (>1,000,000) were collected to ensure adequate detection of integration events. Likewise bookmarking factor knockouts were performed once per guide, but sufficient (n > 30) cells were imaged within each group to ensure data reproducibility. Computational experiments were replicated at least 10 times to determine confidence intervals around estimates. All other experiments (i.e. Live-cell imaging, Hi-C, CRISPRoff) were repeated 2 or more times as independent biological replicates. All replication efforts were successful.
Randomization	All experiments used cultured cell lines. As we were able to directly test the effects of cell treatments (in independent biological replicates), and investigators were not blinded to allocation during experiments and outcome assessment, randomization was not relevant to this study.
Blinding	All data were collected using instruments without bias. Because these data were generated using objective quantifications, researchers assessing results were not blinded for the experimental design. Blinding is not relevant to this study.

Reporting for specific materials, systems and methods

We require information from authors about some types of materials, experimental systems and methods used in many studies. Here, indicate whether each material, system or method listed is relevant to your study. If you are not sure if a list item applies to your research, read the appropriate section before selecting a response.

Materials & experimental systems

n/a	Involved in the study
☐	☒ Antibodies
☐	☒ Eukaryotic cell lines
☒	☐ Palaeontology and archaeology
☒	☐ Animals and other organisms
☒	☐ Clinical data
☒	☐ Dual use research of concern
☒	☐ Plants

Methods

n/a	Involved in the study
☒	☐ ChIP-seq
☒	☐ Flow cytometry
☒	☐ MRI-based neuroimaging

Antibodies

Antibodies used

Aurora B Antibody (Novus Biologicals, NBP2-50039; 1:1000)
CHD1 (Novus Biologicals, NBP2-14478; 1µg/mL, lot 000008248)
HEY1 (Novus Biologicals, NBP2-16818; 1:1000, lot 43097)
SMARCE1 (Sigma-Aldrich, HPA003916; 1µg/mL, lot A107052)
F(ab')2-Goat anti-Rabbit IgG (H+L) Cross-Adsorbed Secondary Antibody, Alexa Fluor™ 488 (Invitrogen, A-11070, 1:500, lot 2896481)
Donkey anti-Mouse IgG (H+L) Highly Cross-Adsorbed Secondary Antibody, Alexa Fluor™ 647 (Invitrogen, A-31571, 1:500, lot 2720365)

Validation

All antibodies were validated by the manufacturers, and are validated to react with corresponding human antigens. Citation data are acquired from CiteAb database:
- Aurora B Antibody (Novus Biologicals, NBP2-50039; 1:1000), 3 citations, validated by manufacturer for immunocytochemistry-immunofluorescence, immunohistochemistry and western blot
- CHD1 (Novus Biologicals, NBP2-14478; 1µg/mL), 0 citations, validated by manufacturer for immunocytochemistry-immunofluorescence, immunohistochemistry and immunohistochemistry-paraffin
- HEY1 (Novus Biologicals, NBP2-16818; 1:1000), 0 citations, validated by manufacturer for immunocytochemistry-immunofluorescence, immunohistochemistry and western blot
- SMARCE1 (Sigma-Aldrich, HPA003916; 1µg/mL), 5 citations, validated by manufacturer for immunocytochemistry-immunofluorescence, immunohistochemistry and western blot
- F(ab')2-Goat anti-Rabbit IgG (H+L) Cross-Adsorbed Secondary Antibody, Alexa Fluor™ 488 (Invitrogen, A-11070, 1:500), 1006 citations
- Donkey anti-Mouse IgG (H+L) Highly Cross-Adsorbed Secondary Antibody, Alexa Fluor™ 647 (Invitrogen, A-31571, 1:500), 2404 citations

Eukaryotic cell lines

Policy information about cell lines and Sex and Gender in Research

Cell line source(s)

The GBM39 cell line was derived from a patient with glioblastoma undergoing surgery at Mayo Clinic, Rochester, Minnesota and was established and obtained as described previously (PMID: 16609043, 28178237). COLO320DM and K562 were obtained from ATCC. GM12878 was obtained from the Coriell Institute for Medical Research.

Authentication

Cell lines obtained from ATCC and Coriell were not authenticated. GBM39 was previously authenticated by the Mischel lab using metaphase DNA FISH as done in Turner et al. 2017 (PMID: 28178237).

Mycoplasma contamination

Cells were tested negative for mycoplasma.

Commonly misidentified lines (See ICLAC register)

None of the cell lines used are registered by ICLAC as commonly misidentified.
